# Supplementary material for: Flower, fruit phenology and flower traits in Cordia boissieri (Boraginaceae) from northeastern Mexico
Source: PeerJ. 2016 May 17;4:e2033. doi: 10.7717/peerj.2033 (PMC4878375; doi:10.7717/peerj.2033)
Supplement: Supplemental Information 1 — pdf file with raw data with explicit headings [file peerj-04-2033-s001.pdf]

STYLE_TYPE	STIGMA_NUM	STAMEN_NUM	TOTAL_LENGT	COROLLA_LEN	SEPAL_LENGT	COROLLA_DIA
2	2	5	26.3	19.8	6.5	34.35
2	2	5	30.65	20.4	10.25	32.65
2	2	5	30.4	18.15	12.25	26.65
2	2	5	27.15	20.75	6.4	37.2
2	2	5	32.9	20.1	12.8	36.95
2	2	5	29.5	22.7	6.8	35.9
2	2	6	19.3	14.65	4.65	35.9
2	2	5	36.85	27.8	9.05	38.05
2	2	5	33.05	18.45	14.6	30.95
2	2	5	31	19.95	11.05	36.4
2	2	5	27.95	21.65	6.3	34.6
2	2	5	33.35	24.65	8.7	36.6
2	2	6	36.6	22.85	13.75	34.7
2	2	5	27.1	16.4	10.7	41.55
2	2	5	26.15	15.3	10.85	32.8
2	2	5	30.3	17.15	13.15	31.45
2	2	5	30.45	20.85	9.6	35.35
2	2	5	30.25	18.3	11.95	36.3
2	2	5	31.6	16.65	14.95	55.9
2	2	5	36.95	29.85	7.1	34.5
2	2	4	29.55	21.6	7.95	35.85
2	2	5	30.3	22.75	7.55	31.6
2	3	5	28.3	19.85	8.45	32.05
2	2	5	33.3	21.95	11.35	26.9
2	2	5	32	22	10	35.6
2	2	5	32.7	23.15	9.55	34.65
2	2	5	28.1	19.85	8.25	33.7
2	3	5	31	20.6	10.4	38.9
2	2	5	32.8	23	9.8	39.6
2	2	6	33	22.5	10.5	35.5
2	2	4	30.25	19.35	10.9	43.15
2	2	5	30	19.7	10.3	43.4
2	2	6	24.85	11.1	13.75	30.45
2	4	5	34.4	24.45	9.95	40.25
2	2	4	28.95	20.75	8.2	35
2	2	4	22.65	13	9.65	42.25
2	2	5	25.4	18	7.4	34.6
2	2	5	30.75	17.95	12.8	37.2
2	2	5	26.3	16.2	10.1	36.7
2	2	5	23	14.1	8.9	39.95
2	2	5	33.45	24.8	8.65	39.25
2	2	5	37.6	25.4	12.2	42.6
2	2	5	29.3	22.05	7.25	31.6
2	2	5	25.1	16.15	8.95	33.05
2	3	5	30.25	23.35	6.9	34.8
2	2	5	31.1	20.4	10.7	28.95

2	2	5	39.15	30.25	8.9	33.3
2	2	5	26.4	15.1	11.3	31
2	2	5	29.25	19.5	9.75	47.5
2	2	5	31.3	20.8	10.5	45.5
2	2	5	31.4	20.9	10.5	37.9
2	2	5	31.6	20.8	10.8	43.7
2	2	5	28.4	18.2	10.2	34.5
2	2	5	33.05	24.6	8.45	39.25
2	2	5	28.5	15.4	13.1	32.9
2	2	5	31.45	21.6	9.85	38.4
2	2	5	32.2	22.75	9.45	36.6
2	2	5	38.8	26.7	12.1	39.95
2	2	5	34.1	22.6	11.5	41.65
2	3	5	39.95	29.95	10	42.15
1	2	5	44	33.8	10.2	65.5
1	2	5	41.95	27	14.95	63.65
1	2	5	44.1	28.2	15.9	60.85
1	2	4	37.2	25.05	12.15	59.7
1	2	5	43.15	29.15	14	64.95
1	2	5	42.7	30.1	12.6	59.95
1	2	5	43.45	30.3	13.15	71.75
1	2	5	42	31.6	10.4	67.4
1	2	6	43.85	29.1	14.75	59.65
1	2	5	43.4	31.35	12.05	61.9
1	2	5	42.2	31.6	10.6	70.75
1	2	5	39.7	27.7	12	54
1	2	4	51.25	35.85	15.4	67.5
1	2	5	45.75	34.5	11.25	73.45
1	2	5	42.85	28.75	14.1	61.5
1	2	5	47.85	34.45	13.4	57.1
1	2	5	45.45	25.3	20.15	59
1	2	5	47.95	32.45	15.5	59.3
1	2	4	47.15	38.1	9.05	60.3
1	2	5	43	27.15	15.85	75
1	2	5	32.5	23	9.5	45.05
1	2	4	27	16.75	10.25	45.1
1	2	5	29	20.15	8.85	49.75
1	2	5	37.8	26.7	11.1	48
1	2	5	37.55	25	12.55	43.75
1	2	5	34.35	21.85	12.5	45
1	2	5	33.7	23.6	10.1	47.6
1	2	5	35.4	26.3	9.1	43
1	2	5	37.3	24.7	12.6	49
1	2	5	38.55	25.7	12.85	51.15
1	2	5	33.45	20	13.45	54.55
1	2	5	38.25	28	10.25	48
1	2	5	39.3	25.4	13.9	52.4

1	2	5	37.5	25.5	12	53.45
1	2	5	33.35	22.45	10.9	48.55
1	2	5	36	23.45	12.55	52.6
1	2	5	37	25.4	11.6	49.85
1	2	5	35.15	24.35	10.8	47.15
1	2	5	41	27.95	13.05	51.05
1	2	4	35.35	23	12.35	52.8
1	2	5	41.8	30.45	11.35	57.45
1	2	5	45.3	32.3	13	58.75
1	2	5	42.45	30.5	11.95	54
1	2	5	44.65	29.4	15.25	52.7
1	2	5	42.95	28.85	14.1	51.5
1	2	5	44.25	27.5	16.75	55.9
1	2	5	44.7	32.6	12.1	45.1
1	2	5	39	28.4	10.6	43.1
1	2	5	49	34.35	14.65	57.6
1	2	5	44.6	32.2	12.4	46.8
1	2	5	42.7	35.55	7.15	53.55
1	2	5	41.5	26.75	14.75	51.8
1	2	5	42.25	31.9	10.35	49.3
1	2	4	43.4	30.6	12.8	53.8
1	2	5	39.7	28.65	11.05	51.5
1	2	5	41.5	29.9	11.6	48.95
1	2	5	45.95	32.1	13.85	51.9
1	2	5	43.7	30.15	13.55	49.6
1	2	4	46.3	30.1	16.2	49.65
1	2	5	45.55	32.4	13.15	60
1	2	5	38.7	27.85	10.85	59.35
1	2	5	41	29.9	11.1	54.95
1	2	5	31.1	18.3	12.8	66.05
1	2	5	35.25	26.1	9.15	56
1	2	5	41.05	27.25	13.8	53.1
1	2	4	34.55	24	10.55	55.45
1	2	5	35.35	22.55	12.8	53.85
1	2	5	35.8	21.45	14.35	60.5
1	2	5	38.25	27.2	11.05	57.15
1	2	5	40.5	29.75	10.75	61.5
1	2	5	35.4	23.05	12.35	60
1	2	5	35.1	23.3	11.8	61.8
1	2	5	40.1	32.75	7.35	60.5
1	2	5	39.9	28	11.9	57.6
1	2	4	39.2	31.45	7.75	49.8
1	2	6	39.25	18.85	20.4	61
1	2	3	30.45	18.3	12.15	43.5
1	2	5	35.15	23.8	11.35	55.4
1	2	5	27.85	18	9.85	57.75
1	2	4	38	27	11	54.35

1	2	5	34.4	25.1	9.3	38.95
1	2	5	35	22.6	12.4	40.55
1	2	5	31.35	19.7	11.65	50.95
1	2	5	31.55	21.25	10.3	44.65
1	2	5	39.65	29.7	9.95	49.3
1	2	6	31.2	22.1	9.1	50.15
1	2	5	37	24.05	12.95	37.2
1	2	5	33.65	23.45	10.2	46
1	2	5	32.8	23.7	9.1	45.85
1	2	5	32.45	24.4	8.05	51.5
1	2	5	29.45	22	7.45	46.2
1	2	5	35	25.9	9.1	43
1	2	5	33.3	24.05	9.25	48.9
1	2	5	31	21.55	9.45	41.05
1	2	5	33.55	25.2	8.35	45.9
1	2	5	34.7	26.2	8.5	48.1
1	2	4	37.85	26.35	11.5	40.15
1	2	5	38.6	26.95	11.65	43
1	2	4	37.7	25.9	11.8	41
1	2	5	36.35	24.55	11.8	41.05
1	2	5	37.3	22.15	15.15	41.4
1	2	5	40.5	28.3	12.2	46.3
1	2	5	41.3	27.95	13.35	35.25
1	2	5	38.95	25.15	13.8	43.35
1	2	5	32.6	24.95	7.65	44.05
1	2	5	33.2	19.95	13.25	47
1	2	5	31.9	19.75	12.15	43.9
1	2	5	32.3	21.7	10.6	46.6
1	2	5	36.15	27.5	8.65	48.95
1	2	5	37.4	25.65	11.75	47.15
1	2	5	40.7	25.45	15.25	46.05
1	2	5	36.05	22.65	13.4	41.7
1	2	5	38.9	27	11.9	48.5
1	2	5	38.8	26.1	12.7	53.7
1	2	4	36.9	21.95	14.95	42.7
1	2	5	41.8	29.35	12.45	54.3
1	2	5	33.3	23.9	9.4	50.15
1	2	5	37.2	23.95	13.25	53.95
1	2	5	30.8	19.55	11.25	53.9
1	2	5	33.95	24.3	9.65	42.9
1	2	5	38.7	25.9	12.8	50.3
1	2	7	41.2	31.7	9.5	61.95
1	2	5	36.2	28.85	7.35	51.5
1	2	6	39.4	23.7	15.7	47.3
1	2	5	38.55	28.95	9.6	52.95
1	2	5	31.85	25.4	6.45	53.95
1	2	5	35.6	22.7	12.9	41.4

1	2	6	38	26.5	11.5	68.45
1	2	6	43.95	34.55	9.4	61.05
1	2	6	34	20.05	13.95	41.15
1	2	5	33.4	24	9.4	52.15
1	2	5	37.45	23.5	13.95	58.1
1	2	5	38.35	28.3	10.05	50.3
1	2	5	35.6	25.15	10.45	52
1	2	6	33.45	28.35	5.1	53.95
1	2	5	32.4	22.5	9.9	48.25
1	2	6	36.1	27.05	9.05	25.4
1	2	5	40.45	29.7	10.75	54.9
1	2	6	35.3	20.65	14.65	66.05
1	2	5	35.3	27.3	8	61
1	2	5	32.45	25.45	7	40.1
1	2	5	34	22.4	11.6	45.15
1	2	5	32.2	20.05	12.15	42.3
1	2	5	37.6	25.25	12.35	38.05
1	2	5	38.8	27.3	11.5	41.6
1	2	5	32.6	19.45	13.15	47.75
1	2	5	38.1	25.4	12.7	38.75
1	2	5	36.1	24	12.1	37.5
1	2	5	39.95	26.65	13.3	39.5
1	2	5	37.1	24.85	12.25	43.75
1	2	5	34.75	25.45	9.3	36.5
1	2	7	34.5	25.9	8.6	42.75
1	2	5	31.45	21.95	9.5	39.85
1	2	5	35.15	25.1	10.05	41.95
1	2	5	37.7	26.6	11.1	36
1	2	5	37.5	25.55	11.95	35.15
1	2	5	39.45	27.75	11.7	39.4
1	2	6	32	24.4	7.6	35.9
1	2	5	32.6	23.8	8.8	44
1	2	5	32.2	25.7	6.5	42.95
1	2	5	34.4	23.6	10.8	45.75
1	2	5	33.4	24	9.4	39.1
1	2	5	38.3	26.6	11.7	43.95
1	2	5	36.5	21.2	15.3	37.35
1	2	5	31.6	20.8	10.8	36.75
1	2	5	33.1	23.95	9.15	39.5
1	2	6	36.7	23.95	12.75	43.25
1	2	6	38.3	26.8	11.5	43.7
1	2	5	32.7	19.45	13.25	42.8
1	2	5	33.6	22.95	10.65	43.95
1	2	5	38.75	25.7	13.05	36.6
1	2	5	38.4	27.25	11.15	43.5
1	2	5	37.35	25.35	12	49
1	2	5	35.4	25.45	9.95	48.35

1	2	5	34.6	24	10.6	44.05
1	2	5	34.4	23.45	10.95	38.4
1	2	5	29.6	17.3	12.3	42.8
1	2	5	35.9	24.1	11.8	40.4
1	2	5	37.9	26.5	11.4	39.1
1	2	6	27.5	17.8	9.7	41.95
2	2	5	29.75	14.75	15	44.95
2	2	5	38.7	22.15	16.55	49.6
2	2	5	28.1	14.25	13.85	53.95
2	2	5	34.4	20.2	14.2	46.65
2	2	5	34.65	21.55	13.1	51.5
2	2	5	35.05	22.25	12.8	47.2
2	2	5	35.1	23.3	11.8	48.05
2	2	5	34.85	23.6	11.25	56.35
2	2	5	31.6	15.9	15.7	48.95
2	2	5	40.6	27.9	12.7	56.45
2	2	5	33.65	21.2	12.45	44.4
2	2	5	29.3	17.25	12.05	51.9
2	2	5	33.9	20.15	13.75	44.4
2	2	5	29.6	17.55	12.05	42.7
2	2	5	39.45	26.25	13.2	46.2
2	2	5	33.6	19.65	13.95	53.05
2	2	5	33.15	20.1	13.05	46.85
2	2	5	31.75	21.85	9.9	55.9
2	2	5	34.2	23.1	11.1	53.05
2	2	5	35.95	22.7	13.25	58.85
2	2	5	39.7	21.85	17.85	50.4
2	2	5	41.35	26.05	15.3	54.15
2	2	5	37.7	27.6	10.1	52
2	2	5	42.05	29.15	12.9	53.45
2	2	5	42.05	28.25	13.8	56
2	2	5	42.35	25.55	16.8	43.1
2	2	5	37.4	26.9	10.5	46.45
2	2	5	38.25	29.9	8.35	40.5
2	2	5	40.1	22.6	17.5	61.1
2	2	5	40.1	26.15	13.95	55.5
2	2	5	40.3	27.45	12.85	58.8
2	2	5	40.55	24.45	16.1	50.85
2	2	5	29.95	16	13.95	48.2
2	2	5	35.95	28.55	7.4	50.45
2	2	5	37.4	19.3	18.1	49.85
2	2	5	35.15	20.15	15	51.5
2	2	5	37.2	25.15	12.05	65
2	2	5	32	23.8	8.2	55.85
2	2	5	33.3	21.1	12.2	57.7
2	2	5	33.4	20.75	12.65	56.85
2	2	5	42.7	20.6	22.1	59.15

2	2	5	37.9	25	12.9	51.5
2	2	5	35.25	30	5.25	60.95
2	2	5	38	23	15	58.95
2	2	5	38.5	20.75	17.75	48.35
2	2	5	38.5	16.45	22.05	46.8
2	2	5	43.05	22.4	20.65	51.05
2	2	5	39.05	22	17.05	53.75
2	2	5	38.1	21.3	16.8	57.55
2	2	5	40.35	25.8	14.55	52.2
2	2	5	35.15	21.9	13.25	50.95
2	2	5	43.1	20.95	22.15	54.95
2	2	5	39.8	21.25	18.55	54.15
2	2	5	43.65	21.1	22.55	51.45
2	2	5	42	26.25	15.75	43.05
2	2	5	38.9	21.1	17.8	43.35
2	2	5	42.6	28.2	14.4	59.95
2	2	5	39.05	20.5	18.55	60.95
2	2	5	47.4	25.85	21.55	50
2	2	5	41.25	19.7	21.55	43.6
1	2	5	38.4	28.55	9.85	58.3
1	2	5	35.65	24.25	11.4	65.1
1	2	5	44.55	27.6	16.95	71.15
1	2	5	40.15	25.8	14.35	72.45
1	2	5	42.9	33.65	9.25	64.05
1	2	5	40	25.7	14.3	66.9
1	2	5	36.3	24.9	11.4	63.95
1	2	5	43.45	28.05	15.4	68.15
1	2	5	43	31.4	11.6	65.65
1	2	5	47.7	33.45	14.25	64.7
1	2	5	35.3	23.55	11.75	61.95
1	2	5	45.6	34.05	11.55	65.5
1	2	5	40.45	27.6	12.85	61.6
1	2	5	38.7	21.7	17	66.9
1	2	5	37.25	20.85	16.4	67.95
1	2	5	47.05	30.55	16.5	60.55
1	2	5	45.4	32.65	12.75	63.85
1	2	5	44.9	31.5	13.4	64.5
1	2	5	38.85	24.75	14.1	63.7
1	2	5	37.1	19.55	17.55	69.95
1	2	5	32.2	16.95	15.25	57.1
1	2	5	29.6	18.2	11.4	43.45
1	2	5	37.6	22.1	15.5	56.95
1	2	5	38.6	25.65	12.95	49.1
1	2	5	30.3	18.05	12.25	53.6
1	2	5	36.65	25	11.65	44.15
1	2	5	35.4	25.05	10.35	51.4
1	2	5	34.6	21.9	12.7	47.95

1	2	5	40.8	26.9	13.9	51.15
1	2	5	36.65	23.8	12.85	50.05
1	2	5	34.7	21.1	13.6	50.35
1	2	5	35.6	20.75	14.85	52.65
1	2	5	31.2	20.6	10.6	47.95
1	2	5	38.2	25	13.2	51.65
1	2	5	41.7	30	11.7	53.85
1	2	5	39.7	23.1	16.6	52
1	2	5	34.7	24.5	10.2	54.45
1	2	5	40.75	28.05	12.7	55.05
1	2	5	38	22.15	15.85	52.15
1	2	5	40.75	27.85	12.9	56.15
1	2	6	44.7	34.5	10.2	73.9
1	2	5	35.1	27.55	7.55	70.05
1	2	5	37.2	23.2	14	66.8
1	2	5	32.6	20.65	11.95	68.95
1	3	5	40.75	29.35	11.4	65.75
1	2	5	35	21.95	13.05	71
1	2	6	46.8	34.7	12.1	75.05
1	2	5	43.2	37.3	5.9	69.85
1	2	5	37.8	21.05	16.75	67.95
1	2	5	34.65	22.5	12.15	68.3
1	2	5	35.8	23.05	12.75	71.95
1	2	5	42.9	22.25	20.65	70.95
1	2	5	42.35	22.5	19.85	73.05
1	2	5	32.25	17.7	14.55	73.55
1	2	5	41	24.75	16.25	71.4
1	2	6	32.45	18.8	13.65	67.9
1	2	5	48.5	33.55	14.95	76.95
1	2	6	32.7	20.3	12.4	77
1	2	5	34.3	19.4	14.9	74.85
1	2	5	34.6	23	11.6	70.95
2	2	5	40	27.75	12.25	59.3
2	2	5	46	29.8	16.2	60.65
2	2	5	28.85	19.3	9.55	62.7
2	2	5	31.25	21.1	10.15	59.65
2	2	5	31.45	22.1	9.35	60
2	2	5	38.2	23.85	14.35	62.2
2	2	5	38	24.25	13.75	62.45
2	2	5	32.5	18.15	14.35	61.7
2	3	4	38	22.25	15.75	54.65
2	2	5	30.65	20.8	9.85	62.5
2	2	5	39.55	25.65	13.9	54.9
2	2	5	31	21.45	9.55	57.3
2	2	5	45	25	20	62.2
2	2	6	30	16.85	13.15	59.9
2	2	5	26.05	16.15	9.9	55.55

2	2	5	37.25	25.75	11.5	66.8
2	2	5	35.95	22.5	13.45	61.85
2	2	6	31.55	24.5	7.05	59.85
2	2	5	32.3	27	5.3	54.5
2	2	5	42.2	29.4	12.8	56
2	2	4	24.6	14.55	10.05	34.6
2	2	5	33.3	25	8.3	38.3
2	2	5	31.65	23.1	8.55	39.5
2	2	5	36.55	27.9	8.65	35.7
2	2	5	30.95	19.7	11.25	33.3
2	2	5	31.6	25.25	6.35	35.65
2	2	5	38.6	28.5	10.1	35.2
2	2	5	33.15	25.05	8.1	40.75
2	2	5	36	25.05	10.95	37.7
2	2	4	31.3	20.95	10.35	34.2
2	2	4	31.75	24	7.75	39.35
2	2	5	32.7	25.7	7	39.65
2	2	5	27.2	17.1	10.1	44
2	2	4	33.4	23.35	10.05	36.4
2	2	5	34.7	27.85	6.85	38.75
2	2	5	30	20.3	9.7	43.1
2	2	5	29.65	18.1	11.55	36.3
2	2	5	25.85	15.7	10.15	40.95
2	2	5	26	16.1	9.9	40.2
2	2	5	35	26	9	37.5
2	2	4	30.65	20.8	9.85	54.65
2	2	5	39.55	25.65	13.9	62.5
2	2	5	31	21.45	9.55	54.9
2	2	5	45	25	20	57.3
2	2	5	30	16.85	13.15	62.2
2	2	6	26.05	16.15	9.9	59.9
2	2	5	37.25	25.75	11.5	55.55
2	2	5	35.95	22.5	13.45	66.8
2	2	5	31.55	24.5	7.05	61.85
2	2	6	32.3	27	5.3	59.85
2	2	5	42.2	29.4	12.8	54.5
2	2	5	24.6	14.55	10.05	56
2	2	4	33.3	25	8.3	34.6
2	2	5	31.65	23.1	8.55	38.3
2	2	5	36.55	27.9	8.65	39.5
2	2	5	30.95	19.7	11.25	35.7
2	2	5	31.6	25.25	6.35	33.3
2	2	5	38.6	28.5	10.1	35.65
2	2	5	33.15	25.05	8.1	35.2
2	3	5	36	25.05	10.95	40.75
2	2	5	37.8	27.9	9.9	52.4
2	2	5	38.65	28.5	10.15	42.85

2	2	5	43.2	31.55	11.65	53.8
2	2	5	40.65	27.25	13.4	44.95
2	2	5	41.6	30.7	10.9	46.6
2	2	5	37.6	28.55	9.05	48.35
2	2	5	40	30.95	9.05	43.4
2	2	5	40.1	30.95	9.15	48.55
2	2	5	39.7	29.35	10.35	42.95
2	2	7	41.35	29.9	11.45	40.25
2	2	5	37.7	26.15	11.55	46.9
2	2	5	42.05	33.4	8.65	53.55
2	2	5	42.05	26.4	15.65	49
2	2	5	42.35	31.6	10.75	46.4
2	2	5	37.4	27.5	9.9	37.6
2	2	5	38.25	26.3	11.95	39.7
2	2	5	40.1	28.6	11.5	44.65
2	2	5	40.1	29.75	10.35	38.7
2	2	5	40.3	29.75	10.55	45.55
2	2	5	40.55	27.2	13.35	48.55
2	2	5	29.95	21.25	8.7	32.7
2	2	5	25.95	15.8	10.15	33.75
2	2	5	37.4	25.5	11.9	41.6
2	2	5	35.15	24.15	11	34.05
2	2	5	37.2	25.25	11.95	34.6
2	2	5	32	22.95	9.05	35.6
2	2	5	33.3	23.75	9.55	40
2	2	5	33.4	24.05	9.35	37.7
2	2	5	37.05	25.4	11.65	34.7
2	2	5	31.8	20.5	11.3	40.25
2	2	5	25.7	17.95	7.75	40.05
2	2	5	31.4	23.7	7.7	36.85
2	2	5	38.4	28.8	9.6	41.5
2	2	4	33.95	24.6	9.35	36.35
2	2	5	34.8	25.7	9.1	39.3
2	2	5	32.5	19.7	12.8	38.95
2	2	5	36.7	25.7	11	40
2	2	5	31.5	22.05	9.45	35.1
2	2	5	37.2	25.3	11.9	39.15
2	2	5	29.4	16.4	13	40.95
2	2	5	39.95	29.4	10.55	47.2
2	2	6	36.7	28.8	7.9	51.2
2	2	4	42	32.6	9.4	43.25
2	2	5	40.25	27.1	13.15	44.6
2	2	5	42.4	31.2	11.2	47.25
2	2	4	35.6	24	11.6	53.7
2	2	6	38.1	29.9	8.2	56
2	2	4	41.95	31	10.95	47.8
2	2	5	36.6	24.65	11.95	49.3

2	2	5	38.7	27.85	10.85	40.9
2	2	5	39.7	31.55	8.15	55.1
2	2	5	37.8	29.6	8.2	48.25
2	2	5	41.5	32.1	9.4	54
2	2	5	43	29.65	13.35	52.4
2	2	5	45.8	30.4	15.4	49.95
2	2	5	42.2	31.75	10.45	52.05
2	2	5	39.1	16.15	22.95	55.05
2	2	5	42.6	29.65	12.95	30.7
2	2	5	39.3	26.05	13.25	53.05
2	2	5	38.95	24.55	14.4	52.6
2	2	5	30	20.3	9.7	42
2	2	5	31.6	20.1	11.5	44.6
2	2	5	33.45	23.7	9.75	47
2	2	5	30.65	20	10.65	48.3
2	2	5	28.85	22	6.85	45.9
2	2	5	36	24	12	42.55
2	2	5	31.2	23.15	8.05	41.5
2	2	5	35	21.15	13.85	51.4
2	2	5	32.35	19	13.35	41.25
2	2	5	34.35	23.6	10.75	44.75
2	2	4	33.1	23.7	9.4	40.5
2	3	5	33.75	24.45	9.3	49.3
2	2	4	29.45	20.3	9.15	44.95
2	2	5	33.45	21.2	12.25	51
2	2	5	33.65	26.6	7.05	50.3
2	2	5	30	18.15	11.85	50.4
2	2	5	32.65	18	14.65	54.15
2	2	4	32.7	19.7	13	52
2	2	4	35.9	23.15	12.75	53.45
2	2	5	34.8	24.65	10.15	56
2	2	5	39	28.55	10.45	43.1
2	2	5	34.75	23.7	11.05	46.45
2	2	5	34.3	26	8.3	40.5
2	2	4	35.4	27.7	7.7	44.8
2	2	6	35.85	25.05	10.8	42.9
2	2	5	36.55	25	11.55	46.1
2	2	5	35.35	25.05	10.3	30.2
2	2	5	35.45	23.55	11.9	42.5
2	2	5	37.4	25.35	12.05	31.2
2	2	5	34.55	24.15	10.4	44.75
2	2	5	32.35	20.2	12.15	44.55
2	2	5	35.6	26	9.6	48.95
2	2	5	34.6	26.45	8.15	42.55
2	2	5	40.55	31.6	8.95	41.5
2	2	5	35.25	27.05	8.2	38.65
2	2	5	26	17	9	40.5

2	2	5	31.35	22	9.35	45
2	2	4	27.5	17.95	9.55	40.25
2	2	4	37.3	28.95	8.35	40.15
2	2	5	37.25	25.85	11.4	45.75
2	2	5	34.35	23.6	10.75	41.25
2	2	4	33.1	23.7	9.4	44.75
2	3	5	33.75	24.45	9.3	40.5
2	2	4	29.45	20.3	9.15	49.3
2	2	5	33.45	21.2	12.25	44.95
2	2	5	33.65	26.6	7.05	51
2	2	5	30	18.15	11.85	50.3
2	2	5	32.65	18	14.65	50.4
2	2	4	32.7	19.7	13	54.15
2	2	4	35.9	23.15	12.75	52
2	2	5	34.8	24.65	10.15	53.45
2	2	5	39	28.55	10.45	56
2	2	5	34.75	23.7	11.05	43.1
2	2	5	34.3	26	8.3	46.45
2	2	4	35.4	27.7	7.7	40.5
2	2	6	35.85	25.05	10.8	44.8
2	2	5	36.55	25	11.55	42.9
2	2	5	35.35	25.05	10.3	46.1
2	2	5	35.45	23.55	11.9	30.2
2	2	5	37.4	25.35	12.05	42.5
2	2	4	29.6	19.75	9.85	52.65
2	2	4	31.25	19	12.25	57.7
2	3	5	33.55	24	9.55	61.55
2	2	4	27.75	21.65	6.1	63.75
2	2	5	27	17.6	9.4	64
2	2	5	30.25	20	10.25	59.05
2	3	5	31.7	22	9.7	60.35
2	2	5	31.65	22.7	8.95	61.95
2	2	6	32.3	19.15	13.15	68.95
2	2	5	30.25	19	11.25	62.05
2	2	4	31.45	21.2	10.25	54
2	2	6	33.25	21.45	11.8	58.7
2	2	5	30	18.45	11.55	61.1
2	2	5	33.2	23.45	9.75	53.95
2	2	4	35.25	21.85	13.4	52
2	2	5	36	26.05	9.95	58.3
2	2	5	27.05	17.6	9.45	58.95
2	3	5	30.05	19.15	10.9	61.4
2	3	5	32.1	18.25	13.85	54.75
2	2	6	35.5	25.55	9.95	66.6
2	2	5	42	26.9	15.1	53.55
2	2	5	41	29.9	11.1	47.85
2	3	5	37.55	22.6	14.95	52.75

2	2	5	38	26.15	11.85	57.45
2	2	5	36.65	27.45	9.2	54.4
2	2	5	37.3	24.45	12.85	56.35
2	2	5	28.3	16	12.3	61.85
2	2	5	40.1	28.55	11.55	53.15
2	2	5	34.2	19.3	14.9	61.55
2	2	5	32.35	20.15	12.2	58.05
2	2	5	36.25	25.15	11.1	58.2
2	2	5	45.55	31.35	14.2	41.95
2	2	5	40.15	29.15	11	48
2	2	5	32.35	21.15	11.2	56.5
2	2	5	38.25	25	13.25	53.35
2	2	6	31.95	22.7	9.25	56.35
2	2	4	30.65	21.9	8.75	42.45
2	2	5	40.45	30.55	9.9	42.6
2	2	5	39.1	31.7	7.4	53.8
2	2	5	31.3	25.75	5.55	48.4
2	2	5	35.8	22.15	13.65	54.9
2	2	5	42.2	29.15	13.05	54.1
2	3	5	39.4	25.7	13.7	45.4
2	2	5	38.6	27.45	11.15	50.25
2	2	5	44.75	30.5	14.25	43.05
2	2	5	45.4	34.3	11.1	46.5
2	2	5	37.5	16.55	20.95	47.55
2	2	5	45.4	33.35	12.05	46.9
2	2	5	32.9	25.7	7.2	41.85
2	2	5	43.45	30.25	13.2	41.4
2	2	5	42.5	31.2	11.3	41.9
2	2	5	43.6	32.9	10.7	38.95
2	2	5	44.05	30.65	13.4	45.05
2	3	5	38.25	26.5	11.75	45.15
2	2	5	39.1	28.05	11.05	40.1
2	2	5	43	28.9	14.1	40.45
2	2	5	39.3	26	13.3	56.1
2	3	5	45.5	31.65	13.85	47.15
2	2	5	41.7	29.7	12	46.1
2	2	5	43.2	30.5	12.7	48.2
1	2	5	41.1	28.55	12.55	60.95
1	2	5	42.95	28.55	14.4	57.7
1	2	5	41.35	27.95	13.4	56.9
1	2	5	29.1	17.55	11.55	53.15
1	2	5	38.85	30.1	8.75	53.75
1	2	5	33.75	22.9	10.85	60.25
1	2	5	43.2	29.55	13.65	57.95
1	2	5	39.95	21.85	18.1	47.55
1	2	5	43.3	32.25	11.05	49
1	2	5	37.9	25.25	12.65	66

1	2	5	45.3	29.4	15.9	49.8
1	2	4	44.8	35.6	9.2	49.65
1	2	5	43	30.65	12.35	58.9
1	2	5	39.3	25.4	13.9	62.05
1	2	5	36.9	26.9	10	52.4
1	2	5	41.1	28.2	12.9	51.65
1	2	5	40.4	29.35	11.05	53.1
1	2	5	42.8	28.95	13.85	57.95
1	2	5	34.6	23.4	11.2	52.45
1	2	5	36.2	25.1	11.1	52.65
1	2	5	51.95	41.1	10.85	64.1
1	2	5	37.5	25.1	12.4	59.8
1	2	4	45.35	34.05	11.3	57.85
1	2	5	41.4	30.05	11.35	60.9
1	2	5	44.05	33.2	10.85	60
1	2	5	41.6	28.65	12.95	61.95
1	2	5	44.95	31.35	13.6	56.1
1	2	5	39.65	27	12.65	60.85
1	2	5	38.1	26.25	11.85	55.8
1	2	5	37.6	24.25	13.35	61.6
1	1	5	42.45	32.05	10.4	53.15
1	2	5	41.3	27.7	13.6	56.05
1	2	5	43.75	31.7	12.05	51.95
1	2	5	39.1	27.15	11.95	60.95
1	2	5	41.4	29.9	11.5	59.5
1	2	5	37.4	24.6	12.8	56.9
1	2	5	34.2	19.65	14.55	57
1	2	5	43.95	30.6	13.35	53.1
1	2	5	39.05	27.6	11.45	46.05
1	2	5	41.1	28.85	12.25	44.55
1	2	5	31.1	19.25	11.85	48.05
1	2	5	34.35	19.55	14.8	63.4
1	2	5	29.75	17.7	12.05	48.3
1	2	5	29.3	17.2	12.1	52.35
1	2	5	38.4	24.8	13.6	43.55
1	2	5	32.8	20.8	12	45.15
1	2	6	32.1	22.1	10	40.1
1	2	4	34.4	20.25	14.15	39.4
1	2	5	34.1	23.15	10.95	52
1	2	5	30.8	19.5	11.3	54.5
1	2	5	31.4	20.45	10.95	44.95
1	2	4	32.7	18.75	13.95	45.2
1	2	4	32.35	19.25	13.1	34.2
1	2	5	27.25	13.4	13.85	48.7
1	2	5	34.2	23.1	11.1	45.35
1	2	5	32	19.05	12.95	47.7
1	2	5	34	20.8	13.2	55.3

1	2	5	34.25	19.25	15	58.4
1	2	5	33.6	19.65	13.95	52.85
1	2	5	37.05	25	12.05	53.05
1	2	5	47.85	34.15	13.7	60
1	2	5	44.4	31.15	13.25	60.75
1	2	4	44.45	32.5	11.95	45.3
1	2	4	49.4	37.15	12.25	46.65
1	2	5	34.75	25.5	9.25	54.65
1	2	5	45.25	33	12.25	62.95
1	2	5	39.45	26.95	12.5	56.9
1	2	5	44.95	33.55	11.4	52.4
1	2	5	39.55	27	12.55	55.05
1	2	5	44.7	29.35	15.35	55.2
1	2	5	36.4	27.7	8.7	61.4
1	2	5	45.5	35.25	10.25	57.11
1	2	5	48.05	34.95	13.1	51
1	2	5	35.75	24.41	11.34	59.95
1	2	3	34.85	21.9	12.95	45.65
1	2	4	36.1	26.75	9.35	60.25
1	2	5	37.55	25.61	11.94	58.75
1	2	5	43.55	29.91	13.64	51.25
1	2	5	50.15	36.35	13.8	58.05
1	2	5	48.31	37	11.31	53.6
1	2	5	37.21	27.65	9.56	51.95
1	2	5	37.55	25.1	12.45	48.65
1	2	5	36.15	23.25	12.9	49.95
1	2	5	38.75	27.6	11.15	47.1
1	2	5	38.5	30.65	7.85	53
1	2	5	36.05	24.1	11.95	55.9
1	2	5	39.05	26.6	12.45	52
1	2	5	38.95	25.35	13.6	60
1	2	5	35.1	22.2	12.9	57.25
1	2	5	39.4	29.25	10.15	51.9
1	2	5	40.4	27	13.4	56.3
1	2	5	38.75	25.7	13.05	54.9
1	2	5	44.2	38.05	6.15	56.35
1	2	5	39.15	29.05	10.1	61.05
1	2	5	40.25	29.65	10.6	57.65
1	2	5	34.2	27.55	6.65	60
1	2	5	41.85	28.55	13.3	54.3
1	2	5	40.25	30.35	9.9	53.05
1	2	5	37	25.2	11.8	51.7
1	2	5	43.55	33.25	10.3	59.3
1	2	5	36.4	27.7	8.7	61.4
1	2	6	45.5	35.25	10.25	57.11
1	2	5	48.05	34.95	13.1	51
1	2	5	35.75	24.4	11.35	59.95

1	2	5	34.85	21.9	12.95	45.65
1	2	4	36.1	26.75	9.35	60.25
1	2	5	37.55	25.6	11.95	58.75
1	2	5	43.55	29.9	13.65	51.25
1	2	5	50.15	36.35	13.8	58
1	2	5	48.3	37	11.3	53.6
1	2	5	37.2	27.65	9.55	51.95
1	2	5	37.55	25.1	12.45	48.6
1	2	5	36.15	23.25	12.9	49.95
1	2	5	38.75	27.6	11.15	47.15
1	2	5	38.5	30.65	7.85	53.05
1	2	5	36.05	24.1	11.95	55.91
1	2	5	39.05	26.6	12.45	52.05
1	2	5	38.95	25.35	13.6	60.05
1	2	5	35.1	22.2	12.9	57.2
1	2	5	39.4	29.25	10.15	51.91
1	2	6	34	22.5	11.5	37.75
1	2	5	27.65	17.85	9.8	37
1	2	5	30.8	19.2	11.6	48.85
1	2	5	35.6	24.55	11.05	44.2
1	2	6	38.4	25.7	12.7	50.25
1	2	5	39.45	31.6	7.85	50.65
1	2	5	36.5	25.7	10.8	49.7
1	2	7	34.5	23.1	11.4	37.9
1	2	5	29.15	21.1	8.05	44.95
1	2	5	28.7	18.5	10.2	42.5
1	2	5	38.8	29.35	9.45	46.95
1	2	4	34.55	21.6	12.95	41.1
1	2	5	37.6	25.8	11.8	46.4
1	2	5	35.2	24.8	10.4	49.15
1	2	6	30.3	12.25	18.05	51.45
1	2	4	38.85	27.9	10.95	43.65
1	2	5	43.85	31	12.85	48.6
1	2	4	35.9	26.1	9.8	43.8
1	2	6	32.95	19.5	13.45	48.85
1	2	5	33.75	22.7	11.05	43.65
1	2	5	36.75	26	10.75	55.4
1	2	5	37.65	27.05	10.6	47.8
1	2	5	27.4	17.5	9.9	39.4
1	2	5	29.75	19.95	9.8	45.9
1	2	5	30.3	18.05	12.25	53.6
1	2	5	36.65	25.05	11.6	44.15
1	2	5	35.4	25.05	10.35	51.45
1	2	5	34.6	21.9	12.7	47.9
1	2	5	40.8	26.9	13.9	51.1
1	2	5	36.65	23.8	12.85	50
1	2	5	34.7	21.1	13.6	50.3

1	2	5	35.6	20.75	14.85	52.65
1	2	5	31.2	20.6	10.6	47.95
1	2	5	38.2	25	13.2	51.65
1	2	5	41.7	30	11.7	53.85
1	2	5	39.7	23.1	16.6	52
1	2	5	34.7	24.5	10.2	54.4
1	2	5	40.75	28.05	12.7	55
1	2	5	38	22.15	15.85	52.1
1	2	5	40.75	27.85	12.9	56.1
1	2	5	31.05	19.2	11.85	41.3
1	2	5	35.3	22.2	13.1	47
1	2	5	34.25	21.05	13.2	46.3
1	2	5	29.8	15.65	14.15	44.6
1	2	5	41.1	28.3	12.8	53.95
1	2	5	40.2	27.7	12.5	55.55
1	2	5	34	23.15	10.85	55.5
1	2	6	32.7	21.7	11	46.05
1	2	5	26.15	16.25	9.9	38.7
1	2	5	28.45	17.9	10.55	44.5
1	2	5	29.7	17.6	12.1	40.35
1	2	5	30.6	19	11.6	37.95
1	2	5	32.5	20.05	12.45	41.8
1	2	5	34.2	25.6	8.6	51.95
1	2	5	32.25	19.2	13.05	39
1	2	5	29.2	17.8	11.4	41.75
1	2	6	34.7	23.2	11.5	61.25
1	2	5	33.3	18.5	14.8	41
1	2	5	34.7	23.4	11.3	53.5
1	2	6	30.4	19.25	11.15	46.65
1	2	5	37.7	23.45	14.25	57
1	2	5	38.4	23.6	14.8	55.8
1	2	5	36	26.95	9.05	56.85
1	2	5	38.55	26.1	12.45	54.05
1	2	5	41.1	29.6	11.5	55.4
1	2	5	39.35	28.1	11.25	55.35
1	2	5	42.4	27.15	15.25	54
1	2	5	40.95	29.55	11.4	49.05
1	2	5	42.45	31.25	11.2	48.9
1	2	5	40.6	30.95	9.65	50.95
1	2	5	42.45	34.1	8.35	52.9
1	2	5	39.05	24.5	14.55	60
1	2	5	36.85	26.6	10.25	54.3
1	2	5	40.1	30.25	9.85	50.9
1	2	5	37.05	26.55	10.5	53.15
1	2	4	42.5	32.95	9.55	54.05
1	2	5	41.25	28.9	12.35	54.3
1	2	5	40.15	28.7	11.45	50.55

1	2	5	38	26.5	11.5	50.3
1	2	5	35.95	25.3	10.65	47.95
1	2	5	30.3	19.2	11.1	39
1	2	4	29.25	21.1	8.15	34.3
1	2	5	39	30.3	8.7	42.35
1	2	5	38.6	27.4	11.2	43.3
1	2	5	39.8	30.7	9.1	43.95
1	2	5	35.1	24.7	10.4	42.7
1	2	5	35.3	24.45	10.85	41.35
1	2	5	37.9	27.55	10.35	44.25
1	2	5	38.95	28.3	10.65	47
1	2	5	35.95	27.1	8.85	42.35
1	2	5	37.15	27.4	9.75	42
1	2	5	34.85	25.7	9.15	38.8
1	2	5	35.7	24.05	11.65	41.55
1	2	5	34.05	25.1	8.95	42.7
1	2	5	37.2	27.15	10.05	41.45
1	2	5	36.1	27.35	8.75	44.8
1	2	5	37.5	26.35	11.15	43
1	2	5	34.25	24	10.25	46.2
1	2	5	35.8	26.1	9.7	48.65
1	2	5	34.05	24.15	9.9	41.1
2	2	4	42.3	30.4	11.9	44.5
2	2	5	43.3	33.75	9.55	46.35
2	2	4	39	29	10	45.3
2	2	4	40	30	10	44.6
2	2	5	44	33	11	51
2	2	5	40.5	27.45	13.05	43.8
2	2	5	42.35	31	11.35	47.6
2	2	5	42.9	30.25	12.65	45
2	2	4	42.45	27.2	15.25	39.45
2	2	4	39.7	30.55	9.15	35.2
2	2	5	40.5	31	9.5	49.9
2	2	5	37	30	7	48
2	2	4	42.2	31	11.2	42.9
2	2	6	48.35	35.75	12.6	46.7
2	2	5	41.15	27.35	13.8	46.5
2	2	4	39.6	29	10.6	53.55
2	2	5	42.25	32.3	9.95	48.35
2	2	5	42.95	30.75	12.2	39.3
2	2	4	37.6	29.4	8.2	36.65
2	2	5	44.55	31.3	13.25	46.3
2	2	5	23.75	13.1	10.65	34.15
2	2	5	23.1	14.1	9	39.95
2	2	5	32.5	19.45	13.05	19.45
2	2	5	26.55	19.3	7.25	48.15
2	2	5	28.1	20.35	7.75	40.8

2	2	5	29.2	12.5	16.7	52.75
2	2	5	22.3	15.7	6.6	49.95
2	2	5	28.1	20.5	7.6	49.3
2	2	4	25.05	13.1	11.95	39.85
2	2	5	25.4	14.25	11.15	44.3
2	2	5	24.15	14.15	10	36.35
2	2	5	26.1	19.4	6.7	47.4
2	2	5	22.75	14.55	8.2	39.15
2	2	5	24.05	15.8	8.25	37.75
2	2	6	25.55	10.1	15.45	16.3
2	2	5	24.4	17.9	6.5	41.35
2	2	4	26.8	19.15	7.65	42
2	2	5	27.25	15.65	11.6	42.4
2	2	5	21.05	12.3	8.75	47.85
2	2	5	21.1	12.8	8.3	37.3
2	2	4	39.7	30.55	9.15	35.2
2	2	5	40.5	31	9.5	49.9
2	2	5	37	30	7	48
2	2	4	42.2	31	11.2	42.9
2	2	6	48.35	35.75	12.6	46.7
2	2	5	41.15	27.35	13.8	46.5
2	2	4	39.6	29	10.6	53.55
2	2	5	42.25	32.3	9.95	48.35
2	2	5	42.95	30.75	12.2	39.3
2	2	4	37.6	29.4	8.2	36.65
2	2	5	44.55	31.3	13.25	46.3
2	2	5	23.75	13.1	10.65	34.15
2	2	5	23.1	14.1	9	39.95
2	2	5	32.5	19.45	13.05	19.45
2	2	5	26.55	19.3	7.25	48.15
2	2	5	28.1	20.35	7.75	40.8
2	2	5	29.2	12.5	16.7	52.75
2	2	5	22.3	15.7	6.6	49.95
2	2	5	28.1	20.5	7.6	49.3
2	2	4	25.05	13.1	11.95	39.85
1	2	5	37.5	23.05	14.45	54.9
1	3	5	32.45	23.15	9.3	54.9
1	2	5	40.55	29.6	10.95	49.15
1	2	5	38.7	26.75	11.95	51.9
1	2	5	43.5	31.85	11.65	53.95
1	2	5	43	33.7	9.3	48.05
1	2	5	44.8	32.1	12.7	51.4
1	2	5	42.7	34.5	8.2	56.1
1	2	5	37.9	29.9	8	46.85
1	2	5	35.25	24.55	10.7	32.55
1	2	5	38	30.05	7.95	50.95
1	2	5	38.5	28.1	10.4	47.7

1	2	5	38.5	32.25	6.25	44.25
1	2	5	43.05	32	11.05	49.25
1	2	5	39.05	28.3	10.75	53.2
1	2	5	38.1	29.5	8.6	44.4
1	2	5	40.35	27.5	12.85	50.25
1	2	5	35.15	25.9	9.25	55.6
1	2	5	43.1	31.05	12.05	48.9
1	2	5	39.8	27.6	12.2	51.85
1	2	5	43.65	32.55	11.1	57.3
1	2	5	42	30.65	11.35	61.2
1	2	5	38.9	29.55	9.35	55.55
1	2	5	42.6	28.9	13.7	59
1	2	5	39.05	27	12.05	52.45
1	2	5	47.4	32.3	15.1	58.25
1	2	5	41.25	28.7	12.55	56.85
1	2	6	37.65	28.75	8.9	57.65
1	2	6	44.3	36.35	7.95	52.6
1	2	5	48.6	37.3	11.3	55.45
1	2	5	41.45	36.6	4.85	62.8
1	2	5	45.1	35.4	9.7	57.65
1	2	5	44.85	34.2	10.65	56.8
1	2	5	42.4	30.35	12.05	59.5
1	2	5	44.1	32.05	12.05	59.1
1	2	5	43.35	33.05	10.3	52.8
1	2	5	45	34.95	10.05	60.6
1	2	5	36.5	23.5	13	52.75
1	2	5	34.3	23.45	10.85	54
1	2	5	48.3	35.5	12.8	62.15
1	2	5	46	36.4	9.6	58.05
1	2	5	45.4	28.95	16.45	61.45
1	2	5	47.8	32.7	15.1	53.8
1	2	5	45.25	36.35	8.9	52.2
1	2	5	34.4	24.3	10.1	47.95
1	2	5	45.25	32.25	13	52.9
1	2	5	44	31.7	12.3	58.65
1	2	5	38.7	26.2	12.5	55.35
1	2	5	40.2	30.5	9.7	48.25
1	2	5	39.7	26.6	13.1	56
1	2	5	40.25	28.15	12.1	57.05
1	2	5	43.85	32.4	11.45	57.95
1	2	5	41.2	29.25	11.95	51.05
1	2	5	39.5	30.1	9.4	49.05
1	2	5	39.8	26.1	13.7	55.1
1	2	5	31.1	23	8.1	47.9
1	2	5	37.1	16.6	20.5	43.5
1	2	5	36.7	27.7	9	58.9
1	2	5	39.3	28.55	10.75	52.5

1	2	4	44	28.7	15.3	51.05
1	2	4	41.4	33.25	8.15	65.55
1	2	5	45	31	14	67.2
1	2	4	47.4	31.15	16.25	62.9
1	2	4	37.45	23.5	13.95	63.75
1	2	5	31	17.7	13.3	65.75
1	2	5	40.55	32.9	7.65	63
1	2	5	41.9	27.85	14.05	60.15
1	2	4	38.1	21	17.1	61.55
1	2	4	35	19.75	15.25	60.45
1	2	5	31.7	26.5	5.2	69.6
1	2	5	33.45	20.65	12.8	63.5
1	2	5	35.05	21.9	13.15	69.5
1	2	5	37.85	24.15	13.7	71
1	2	5	40.5	23.15	17.35	70.75
1	2	5	30.6	22.3	8.3	67.45
1	2	5	35	22.45	12.55	66.45
1	2	5	32.45	20.8	11.65	67.45
1	2	5	35.2	21.35	13.85	58.3
1	2	5	36.6	24.75	11.85	65.25
1	2	5	36.35	20.65	15.7	71
1	2	5	37.2	29.3	7.9	55
1	2	5	40.45	27.65	12.8	59.55
1	2	5	41.1	29	12.1	60
1	2	5	38.65	26.75	11.9	63.65
1	2	5	35.6	22.45	13.15	60
1	2	5	33.25	19.4	13.85	53.5
1	2	2	35	26.2	8.8	52.25
1	2	5	39.35	24.8	14.55	57.95
1	2	5	32.45	23.55	8.9	47.7
1	2	5	30.8	18.15	12.65	47.2
1	2	5	20.85	16.4	4.45	45.15
1	2	5	33.85	25.25	8.6	52
1	2	5	44.3	35.8	8.5	58.5
1	2	5	32.45	23	9.45	43.85
1	2	5	31.35	20.75	10.6	49.5
1	2	5	25.5	16.45	9.05	48.05
1	2	5	37.9	22.4	15.5	63
1	2	5	28.75	22	6.75	58
1	2	5	33.2	21.3	11.9	49.05
1	2	5	36.15	25.8	10.35	49.95
1	2	5	44.1	31.9	12.2	57.85
1	2	5	35	20.95	14.05	54.95
1	2	5	58.9	38.6	20.3	63.95
1	2	5	36.7	26.6	10.1	56.9
1	2	5	43.9	33.65	10.25	60.25
1	2	5	44.35	34.1	10.25	59.95

1	2	5	40.9	27.6	13.3	66.7
1	2	5	53.8	40.15	13.65	68.55
1	2	5	35.25	20.25	15	55.8
1	2	5	31.9	17.85	14.05	50.4
1	2	5	32.7	20.55	12.15	52.9
1	2	5	30.8	18.95	11.85	50.9
1	2	5	33.6	21.3	12.3	52.7
1	2	5	42.7	31.7	11	67.6
1	2	5	37.3	23.8	13.5	59.2
1	2	5	46.7	32.9	13.8	62.2
1	2	5	48.3	29.9	18.4	64.95
1	2	5	33.2	20.3	12.9	56.75
1	2	5	38.5	30.75	7.75	60.7
1	2	5	45.05	30.95	14.1	63.9
2	2	5	25.25	15.95	9.3	52.55
2	2	5	32	22.4	9.6	48.05
2	2	5	31.45	20.9	10.55	46.6
2	2	5	35.7	26.7	9	51
2	2	5	42.85	31	11.85	46.9
2	3	6	31	23.1	7.9	47.7
2	2	5	26.3	14.5	11.8	53.15
2	2	5	35.9	23.45	12.45	44.95
2	2	5	27.95	14.9	13.05	51.6
2	2	5	31.65	23.35	8.3	50.5
2	2	6	34.2	25.75	8.45	55.05
2	2	5	35.65	25.1	10.55	48.5
2	2	4	32.4	26.1	6.3	51.55
2	2	5	33.45	18.85	14.6	52.7
2	2	5	33.05	23	10.05	55.05
2	2	5	24.75	13.4	11.35	52.1
2	2	5	24.45	16.2	8.25	52.8
2	2	5	33.85	20.8	13.05	47.95
2	2	5	33.05	24.15	8.9	43.05
2	2	5	34.85	24.75	10.1	53.15
2	2	5	39.5	26.3	13.2	46.9
2	2	5	37	25.35	11.65	47.95
2	2	5	36.4	26.55	9.85	59.3
2	2	5	34.1	23.8	10.3	50.25
2	2	5	34.75	22.95	11.8	46.1
2	2	5	34.8	18.35	16.45	44.95
2	2	5	33.05	24.8	8.25	50.85
2	2	6	34.1	23.25	10.85	51.9
2	2	5	32.6	24.1	8.5	43.7
2	2	5	33.2	25.95	7.25	53.6
2	2	5	33.6	22.75	10.85	48.7
2	2	5	36.85	28.1	8.75	51.2
2	2	6	30.85	22.7	8.15	48.1

2	2	5	36	23.3	12.7	52.05
2	2	6	38.65	28.45	10.2	48.7
2	2	5	36.05	26.8	9.25	51.6
2	2	5	33.95	24.4	9.55	47.9
2	2	5	35.05	22.5	12.55	54.95
2	2	6	35.7	22.6	13.1	54.95
2	2	6	36.35	27.4	8.95	55
2	2	6	28.8	16.35	12.45	38.3
2	2	5	39.1	25.6	13.5	50.85
2	2	5	37.8	25.35	12.45	40.3
2	2	5	42.2	30	12.2	44.3
2	2	5	28.65	18.95	9.7	37.2
2	2	5	42.05	28.2	13.85	38.1
2	2	5	24	12.2	11.8	38.85
2	2	5	34.75	24.75	10	53.1
2	2	6	31.4	18.2	13.2	38.6
2	2	5	38.7	27.7	11	44.55
2	2	5	37.6	19.15	18.45	46.95
2	2	5	25.1	13.7	11.4	35.7
2	2	5	38.5	24.4	14.1	42.55
2	2	5	33.45	22.8	10.65	48.95
2	2	5	33.6	21.9	11.7	51.2
2	2	6	31.3	19.1	12.2	59
2	2	6	34	20.7	13.3	46.3
2	2	5	36.8	24.7	12.1	50
2	2	6	25.2	16.4	8.8	43.6
2	2	6	25.4	16.95	8.45	58
2	2	5	44.4	30.8	13.6	62.2
2	2	5	37.4	27.05	10.35	59.95
2	2	5	42.45	30.5	11.95	68.15
2	2	5	31.6	20.9	10.7	61.05
2	2	5	36.25	26.9	9.35	65
2	2	5	38.05	23	15.05	67.95
2	2	5	34.15	17.8	16.35	67.3
2	2	5	39.4	25.5	13.9	67.8
2	2	5	46.3	33.35	12.95	70.2
2	2	6	43.45	30.4	13.05	64.9
2	2	5	41.5	28.8	12.7	60.75
2	2	6	38.45	27.4	11.05	69.8
2	2	5	36	20.1	15.9	63.1
2	2	5	46.3	31.5	14.8	65.15
2	2	5	42.25	31.6	10.65	62.75
2	2	4	40.85	27.3	13.55	60
2	2	5	34.1	24.7	9.4	60.25
2	2	5	34.3	24.85	9.45	63.1
2	2	5	28.8	19.6	9.2	65.4
2	2	5	48	38	10	64.65

2	2	5	39.1	24.2	14.9	61.1
2	2	5	34.95	24.95	10	55.5
2	2	5	47.55	34.7	12.85	58.8
2	2	5	40.25	32.2	8.05	50.85
2	2	5	38.7	30.3	8.4	48.2
2	2	5	37.15	28.2	8.95	50.45
2	2	5	36.5	28	8.5	49.85
2	2	5	33.7	27.95	5.75	42
2	2	5	36.7	19.35	17.35	52.2
2	2	5	39.95	29.95	10	50
2	2	5	38.4	30.25	8.15	50.55
2	2	5	41.35	26	15.35	51.55
2	2	5	53.45	23.95	29.5	54.15
2	2	5	37.9	25.3	12.6	58.4
2	2	5	31.7	19.05	12.65	57.95
2	2	5	36.05	21.35	14.7	56.35
2	2	5	41.2	27.65	13.55	50.65
2	2	5	38.25	24.4	13.85	49.2
2	2	5	29.7	18.1	11.6	57.8
2	2	5	39.8	26.85	12.95	63
2	2	5	46.45	32.6	13.85	52.8
2	2	5	40.8	30.9	9.9	50.95
2	2	5	35.8	25.9	9.9	54.95
2	2	6	45.65	31.8	13.85	54.15
2	2	5	49.95	35.8	14.15	51.45
2	2	5	33.55	22.25	11.3	43.05
2	2	5	35.3	23.2	12.1	43.35
2	2	5	44.65	29.65	15	59.95
2	2	5	48.85	35.4	13.45	60.95
2	2	4	44.45	32.7	11.75	57.1
2	2	5	46.5	32.75	13.75	51.35
2	2	5	32	18.65	13.35	55.2
2	2	5	32	20.4	11.6	49.15
2	2	6	34.1	24.8	9.3	59
2	2	5	36.4	22.85	13.55	66
2	2	5	33.35	24	9.35	50.1
2	2	5	36.75	21.15	15.6	60.55
2	2	5	37.3	26.6	10.7	49
2	2	5	36.3	21.7	14.6	55.3
2	2	6	33.95	20.5	13.45	61.55
2	2	5	29.55	22.25	7.3	34.35
2	2	5	34.15	25.1	9.05	32.65
2	2	5	36.35	15	21.35	26.65
2	2	6	34.5	25.4	9.1	37.2
2	2	5	34.2	24.9	9.3	36.95
2	2	5	33.25	23.4	9.85	35.9
2	2	5	32	20.35	11.65	35.9

2	2	5	36.1	25.4	10.7	38.05
2	2	5	32.5	22.9	9.6	30.95
2	2	5	33.35	18.9	14.45	36.4
2	2	5	34.05	25.2	8.85	34.6
2	2	5	33.55	23.55	10	36.6
2	2	5	32.6	22.4	10.2	34.7
2	2	5	33.6	24.1	9.5	41.55
2	2	5	36.85	25.4	11.45	32.8
2	2	5	34.8	25.6	9.2	31.45
2	2	5	35.2	24.85	10.35	35.35
2	2	5	33.9	23.1	10.8	36.3
2	2	5	31.5	23.8	7.7	55.9
2	2	5	33.05	21.95	11.1	34.5
2	2	5	32.35	24.25	8.1	35.85
2	2	5	32.7	23.9	8.8	31.6
2	2	5	31.3	20.95	10.35	32.05
2	2	5	29.7	20.75	8.95	26.9
2	2	5	33.95	23.5	10.45	35.6
2	2	5	33.7	24.25	9.45	34.65
2	2	5	34.65	21.1	13.55	33.7
2	2	5	32.35	22.95	9.4	38.9
2	2	5	32.65	23.6	9.05	39.6
2	2	5	33.8	21.65	12.15	35.5
2	2	5	31.7	21.7	10	43.15
2	2	5	37.15	27.5	9.65	43.4
2	2	5	33.85	22	11.85	30.45
2	2	5	33.2	22.05	11.15	40.25
2	2	5	33.25	23.45	9.8	35
2	2	5	34.2	27.5	6.7	42.25
2	2	5	31.8	21.45	10.35	34.6
2	2	5	38.85	26.45	12.4	37.2
2	2	5	33.5	24.7	8.8	36.7
2	2	5	34.5	21.15	13.35	39.95
2	2	5	41.95	31.2	10.75	39.25
2	2	5	36.5	25.5	11	42.6
2	2	5	28	17.4	10.6	31.6
2	2	5	28.75	18.7	10.05	33.05
2	2	5	38.1	27.5	10.6	34.8
2	2	5	30.55	20.6	9.95	28.95
2	2	5	31.1	20.35	10.75	33.3
2	2	5	30.6	21.2	9.4	31
2	2	5	37	27.15	9.85	47.5
2	2	5	34.6	24.85	9.75	45.5
2	2	5	34.15	20.35	13.8	37.9
2	2	5	41.35	28	13.35	43.7
2	2	5	31.35	21.4	9.95	34.5
2	2	5	39.3	29.5	9.8	39.25

2	2	5	36.55	27.05	9.5	32.9
2	2	5	34.65	25.3	9.35	38.4
2	2	5	29.9	20.95	8.95	36.6
2	2	5	34.8	25.95	8.85	39.95
2	2	5	35	23.85	11.15	41.65
2	2	5	34.3	23.65	10.65	42.15
2	2	5	39.1	24.75	14.35	61.15
2	2	5	42.5	29.4	13.1	60.2
2	2	5	39.55	32.1	7.45	64.9
2	2	5	40.25	31.35	8.9	57.9
2	2	5	40.1	30.45	9.65	65.95
2	2	5	43.8	28.65	15.15	60.55
2	2	5	45.2	35.85	9.35	62.75
2	2	5	46.7	32.45	14.25	61.55
2	2	5	44.6	32.7	11.9	58.3
2	2	5	39.45	28.3	11.15	56.5
2	2	5	39.35	29.5	9.85	55.5
2	2	5	43	32.35	10.65	61.9
2	2	5	41.4	29.55	11.85	62.2
2	2	5	27.55	16.1	11.45	66.9
2	2	5	40.95	29.5	11.45	70.4
2	2	5	37.7	28	9.7	66.2
2	2	5	40.95	29.95	11	65.25
2	2	5	36.1	24.65	11.45	59.35
2	2	5	41.05	26.85	14.2	70.65
2	2	5	32.35	20.85	11.5	66.5
2	2	5	31.95	22.4	9.55	49.8
2	2	5	32.3	24.4	7.9	60.15
2	2	5	31.6	23.3	8.3	55.9
2	2	5	32.65	24.05	8.6	58.9
2	2	5	40.1	29.4	10.7	51.7
2	2	5	32	20.95	11.05	61.4
2	2	5	33.8	24.2	9.6	57.85
2	2	5	31.1	20.4	10.7	56.95
2	2	5	27.5	14.4	13.1	58.5
2	2	5	33.3	24.65	8.65	58.95
2	2	5	38.1	28.65	9.45	48.35
2	2	5	40.65	28.5	12.15	46.8
2	2	5	29.65	19.25	10.4	51.05
2	2	5	38.8	29.4	9.4	53.75
2	2	5	30.5	18.5	12	57.55
2	2	5	27.95	17.95	10	52.2
2	2	5	35.1	22.65	12.45	56.2
2	2	5	26.05	15.8	10.25	53.95
2	2	5	33.1	21.25	11.85	59.65
2	2	5	34.6	26.95	7.65	52.95
2	2	5	34.9	24.8	10.1	43.35

2	2	5	43.3	28.5	14.8	65.3
2	2	5	35.1	24.7	10.4	55.55
2	2	5	38.1	27.6	10.5	59.95
2	2	5	45.55	34.45	11.1	63.6
2	2	5	48.15	37.95	10.2	55.95
2	2	5	40.35	30.35	10	57.1
2	2	5	44.55	32.5	12.05	54.9
2	2	5	44.4	34.15	10.25	55.8
2	2	5	39.8	30.45	9.35	61
2	2	5	35	26	9	48.65
2	2	5	32.65	21.4	11.25	45.7
2	2	5	35.55	24.45	11.1	52.9
2	2	5	38.85	29	9.85	52.7
2	2	5	44.35	31.9	12.45	57.1
2	2	5	38.5	27.6	10.9	53.25
2	2	5	45.4	33.8	11.6	53.25
2	2	5	41.9	29.6	12.3	54
2	2	5	39.65	29.05	10.6	50.95
2	2	5	33.7	24.8	8.9	65.75
2	2	5	34.7	23.1	11.6	39.05
2	2	5	31.65	18.75	12.9	43.75
2	2	5	26.35	14.45	11.9	38.8
2	2	5	38.3	27.7	10.6	45.6
2	2	5	27.4	18.05	9.35	40.5
2	2	5	24.15	13.15	11	43.3
2	2	5	30.5	21.4	9.1	42.4
2	2	5	29.95	17.3	12.65	43.9
2	2	6	31.8	18.7	13.1	47.05
2	2	5	30.25	20.9	9.35	37.65
2	2	5	31.35	24.15	7.2	39.6
2	2	5	28.5	18.65	9.85	37.6
2	2	5	29.7	18.95	10.75	39.1
2	2	5	30.85	21.6	9.25	36.25
2	2	5	39.65	20	19.65	38.5
2	2	5	39.95	24.55	15.4	41.75
2	2	5	32.6	22.8	9.8	39.1
2	2	5	31.7	21.6	10.1	40.75
2	2	5	35.15	25.3	9.85	42.3
2	2	5	29.25	17	12.25	44.15
1	2	5	40.25	28.85	11.4	55.05
1	2	5	34.25	22.1	12.15	51.1
1	2	5	36.35	25.35	11	53.5
1	2	5	37.2	24.5	12.7	57.85
1	2	5	38.6	28.56	10.04	56.55
1	2	5	36.65	25.6	11.05	59.4
1	2	5	32.3	20.85	11.45	52.7
1	2	5	37.15	25.3	11.85	53.05

1	2	5	37.2	25.85	11.35	55.45
1	2	5	43.35	29.45	13.9	54.1
1	2	5	37.25	28	9.25	45.9
1	2	5	41.45	28.75	12.7	53.5
1	2	5	36.9	23.8	13.1	40
1	2	5	43.8	33.9	9.9	50.95
1	2	5	37.7	27.2	10.5	55.6
1	2	5	41	29.15	11.85	48.05
1	2	5	43.25	31.95	11.3	48.9
1	2	5	37.15	25.45	11.7	52
1	2	5	38.4	25.35	13.05	54.55
1	2	5	43.45	30.65	12.8	42.95
1	2	5	40.35	25.45	14.9	57.75
1	2	5	40.5	27.7	12.8	60.85
1	2	5	37.4	25.95	11.45	57.55
1	2	5	32.3	21.45	10.85	46.35
1	2	5	34.5	23.35	11.15	50
1	2	5	39.45	24.5	14.95	54.95
1	2	5	38.4	28	10.4	58.05
1	2	5	37.2	30.55	6.65	48.4
1	2	5	32.8	22.45	10.35	57.4
1	2	5	41.85	29.8	12.05	57.5
1	2	5	37.15	31.45	5.7	63.2
1	2	5	44.3	31.8	12.5	67.95
1	2	5	27.6	15.9	11.7	56.6
1	2	5	37.45	27.1	10.35	65.95
1	2	5	35.6	25.25	10.35	61.3
1	2	5	41.6	28.95	12.65	62.45
1	2	5	42.7	30.75	11.95	55.5
1	2	5	40.4	28.45	11.95	61.9
1	2	5	43.1	33.8	9.3	61.9
1	2	5	36.6	27.4	9.2	56.95
1	2	5	32	22.2	9.8	53.05
1	2	5	35.8	21.5	14.3	48.55
1	2	5	38	26.3	11.7	55.6
1	2	5	32.95	21.05	11.9	59.1
1	2	5	34.15	23.25	10.9	47.6
1	2	5	38.05	26.8	11.25	47.9
1	2	5	31.3	21.25	10.05	49.1
1	2	5	32.8	21.1	11.7	45.65
1	2	5	39.1	26.25	12.85	62.7
1	2	5	31.45	21.1	10.35	50.7
1	2	5	40.45	28.2	12.25	66.5
1	2	5	30.95	20.5	10.45	56.2
1	2	5	36.4	25.85	10.55	53.3
1	2	5	34.25	24.15	10.1	63.3
1	2	5	27.2	20.65	6.55	52.2

1	2	5	40.9	28.8	12.1	60.4
1	2	5	31.9	19.75	12.15	59.55
1	2	5	25.15	15.7	9.45	44.45
1	2	5	35.15	22.65	12.5	47.2
1	2	5	33.05	23.05	10	63.15
2	2	5	30.2	18.15	12.05	42.8
2	2	5	28.45	17.4	11.05	44
2	2	4	31.55	22.15	9.4	46.5
2	2	5	33	23.05	9.95	43.35
2	2	5	26.4	17.05	9.35	35
2	2	5	24.3	14.15	10.15	47.4
2	2	5	22.3	14.05	8.25	38.35
2	2	5	36.7	23.05	13.65	49
2	2	5	25.7	18.45	7.25	41
2	2	5	28.2	21.6	6.6	39.25
2	2	5	29.7	23.4	6.3	38.35
2	2	5	28.95	17.05	11.9	39.4
2	2	5	30.95	15.7	15.25	44
2	2	5	27.25	17.15	10.1	42.35
2	2	5	28.25	20.05	8.2	42.05
2	2	5	28.35	22.6	5.75	39
2	2	5	30.95	17.5	13.45	39.95
2	2	5	33.15	20.7	12.45	38.45
2	2	5	23.65	15.65	8	41.95
2	2	5	29.2	20.25	8.95	37.1
2	2	5	35.1	21.9	13.2	58.35
2	2	5	39.5	32	7.5	57.65
2	2	5	41.4	27.85	13.55	52.4
2	2	5	30.7	19.9	10.8	51.5
2	2	5	25.4	15.35	10.05	65
2	2	5	33.8	25.85	7.95	55.85
2	2	5	36	25	11	57.7
2	2	5	24.65	16.75	7.9	56.85
2	2	5	43.4	31.05	12.35	59.15
2	2	5	37.1	23.5	13.6	51.5
2	2	5	34.65	18.55	16.1	60.95
2	2	5	35	21.55	13.45	60.35
2	2	5	28.5	15.15	13.35	59
2	2	5	33.85	23.4	10.45	59.85
2	2	5	32.8	23.5	9.3	60.2
2	2	5	34.95	21.1	13.85	55.15
2	2	5	38.5	25.2	13.3	59.8
2	2	5	41.5	31.55	9.95	59.1
2	2	5	39	25.5	13.5	59.6
2	2	5	34.5	26.3	8.2	53.4
2	2	5	32.3	21.6	10.7	55.8
2	2	5	39.7	24.8	14.9	59.5

2	2	5	38.55	29	9.55	59.9
2	2	5	44.15	31.6	12.55	59.95
2	2	5	41.8	27.8	14	70.3
2	2	5	39.25	25.8	13.45	54.1
2	2	5	36.75	20.65	16.1	47.9
2	2	5	32.6	23.95	8.65	51.6
2	2	5	32.7	18.7	14	49.15
2	2	5	33	22.35	10.65	49.85
2	2	5	34.75	23.85	10.9	49
2	2	5	33.3	22.35	10.95	53.9
2	2	5	36.8	29.35	7.45	57.3
2	2	5	36.65	24.3	12.35	52.6
2	2	5	35.15	26.6	8.55	54.9
2	2	5	33.35	20.55	12.8	55.85
2	2	5	36	23.2	12.8	53.7
2	2	5	37.1	20.75	16.35	55.7
2	2	5	37.55	25.6	11.95	52.1
2	2	5	37.5	25.15	12.35	47.05
1	2	5	31.85	23.4	8.45	55.05
1	2	5	38.25	27.55	10.7	55
1	2	5	36.4	26.25	10.15	60.85
1	2	5	35.75	22.9	12.85	60.95
1	2	5	37.3	25.55	11.75	61.55
1	2	5	41	24.35	16.65	60.85
1	2	5	34.35	25.7	8.65	46.6
1	2	5	41.9	33.2	8.7	57.7
1	2	5	37.9	25.6	12.3	53.65
1	2	5	41.6	32.6	9	57
1	2	5	37.3	30.15	7.15	58.6
1	2	5	39.95	25.65	14.3	63.95
1	2	5	40.2	31.95	8.25	59
1	2	6	41.25	29.05	12.2	69.45
1	2	5	32.7	21.8	10.9	58.05
1	2	5	34.5	21.7	12.8	58
1	2	5	41.2	30.7	10.5	56.9
1	2	5	36.2	27.7	8.5	57.95
1	2	5	38.4	27.7	10.7	56.95
1	2	5	34	21.7	12.3	56.9
1	2	5	37.5	24.35	13.15	56.35
1	2	5	35.7	25.15	10.55	49.8
1	2	5	35.55	23.15	12.4	53
1	2	5	35.7	24.9	10.8	49.6
1	2	5	42.45	28.75	13.7	51.6
1	2	5	39	28	11	50.15
1	2	5	41.55	31.05	10.5	47.35
1	2	5	32.75	23.25	9.5	54.25
1	2	5	40.45	28.25	12.2	46.4

1	2	5	37.35	24.9	12.45	50.55
1	2	5	34.7	24.25	10.45	49.6
1	2	5	37.6	27.95	9.65	51.5
1	2	5	38.7	29.35	9.35	46.95
1	2	5	36.15	22.45	13.7	55.95
1	2	5	39.45	23.7	15.75	51.6
1	2	5	36.05	26.05	10	52.05
1	2	5	32.65	22.6	10.05	48.7
1	2	5	40.35	28.9	11.45	42.3
1	2	5	30	22.95	7.05	47.95
1	2	5	33.6	23.55	10.05	55.65
1	2	5	37.8	25.35	12.45	50.95
1	2	5	42.25	28.05	14.2	54.9
1	2	5	33.3	24.25	9.05	53.25
1	2	5	38.1	25.9	12.2	54.8
1	2	5	37.35	26.5	10.85	51.2
1	2	5	39.55	25.25	14.3	47.2
1	2	5	39.8	29.1	10.7	55.9
1	2	5	46.65	28.6	18.05	51.5
1	2	5	35.95	23.2	12.75	50.35
1	2	5	33.6	18.95	14.65	54
1	2	5	33.6	24.6	9	53.55
1	2	5	37.5	25.75	11.75	48.95
1	2	5	32	21.05	10.95	43.05
1	2	5	43.7	26.1	17.6	58.15
1	2	5	27.7	16.7	11	53.95
1	2	5	39	26.3	12.7	57.3
1	2	5	31.7	22.45	9.25	52.35
1	2	5	30.05	17.6	12.45	51.1
1	2	5	28.9	17	11.9	44.1
1	2	5	34	22.8	11.2	49.9
2	2	5	28.05	16.6	11.45	62.2
2	2	5	41.9	28.3	13.6	54
2	2	5	42	27.95	14.05	57.5
2	2	5	35.35	25.6	9.75	53.05
2	2	5	41.2	30.65	10.55	58.1
2	2	5	41.6	29.5	12.1	59
2	2	5	45.95	26.8	19.15	56.7
2	2	5	36.25	23.05	13.2	63.35
2	2	5	36.3	28	8.3	59.8
2	2	5	43.35	30.35	13	57.3
2	2	5	39.95	26.85	13.1	61.7
2	2	5	36.95	24.45	12.5	53
2	2	5	43.7	27.05	16.65	61.8
2	2	5	44.7	29.25	15.45	63.95
2	2	5	39.95	26.95	13	58.45
2	2	6	35.3	27.85	7.45	60.25

2	2	5	42.65	26.15	16.5	53.2
2	2	5	40.3	27.35	12.95	52.2
2	2	5	41.05	27.25	13.8	57.45
2	2	5	39.05	26.8	12.25	54.05
2	2	5	39.25	25.7	13.55	45.6
2	2	5	37.25	23.6	13.65	45.2
2	2	3	36.5	25.4	11.1	32.3
2	2	5	39.75	24.8	14.95	43.05
2	2	5	31.45	19.9	11.55	37.2
2	2	5	38.95	25	13.95	47.95
2	2	6	36.5	25.2	11.3	54.35
2	2	5	39.5	30.05	9.45	47.6
2	2	5	33.8	20.5	13.3	48
2	2	5	32.3	23.25	9.05	50.55
2	2	6	32	19.75	12.25	47.65
2	2	5	41.95	29.35	12.6	45.15
2	2	6	35.3	24.35	10.95	49
2	2	6	37	26.6	10.4	48.2
2	2	5	26.6	15.65	10.95	45.7
2	2	5	33.7	22.2	11.5	45.75
2	2	4	27.05	16.3	10.75	37.65
2	2	6	37.35	23.55	13.8	51.8
2	2	5	36.7	23.8	12.9	37.9
2	2	4	31.2	24.35	6.85	41.3
2	2	5	35.45	26.8	8.65	35.75
2	2	5	35.4	25	10.4	39.4
2	2	5	35.6	27.35	8.25	30.8
2	2	5	28.8	19.55	9.25	31.45
2	2	5	35.3	25.9	9.4	34.75
2	2	5	34.1	25.9	8.2	38.05
2	2	5	34.05	23.25	10.8	35.3
2	2	5	29.85	21.6	8.25	40.65
2	2	5	36.05	23.1	12.95	34.3
2	2	5	34.9	27.2	7.7	33.6
2	2	5	33.2	24.15	9.05	31.6
2	2	5	29.5	20.25	9.25	33.15
2	2	5	33.4	23.8	9.6	34.5
2	2	5	35.85	23.8	12.05	37.6
2	2	5	38.1	26.5	11.6	36.9
2	2	5	31.3	24.45	6.85	35.45
2	2	5	38.25	26.8	11.45	37.95
2	2	5	33.05	22.95	10.1	37.05
2	2	5	38.05	27.05	11	37
2	2	5	37.15	25.7	11.45	37.7
2	2	5	34.9	23.4	11.5	42.5
2	2	5	35.4	22.9	12.5	42.3
2	2	5	37.35	24.5	12.85	45.05

2	2	5	37.2	25.55	11.65	44.9
2	2	5	36.65	26.35	10.3	40.7
2	2	5	35.1	24.55	10.55	33.3
2	2	5	36	22.3	13.7	42.15
2	2	5	38.6	25.85	12.75	41.75
2	2	5	38.5	24.6	13.9	45.65
2	2	5	37.05	25.9	11.15	42.35
2	2	5	36.9	27.3	9.6	37.5
2	2	5	35.7	22.3	13.4	45.95
2	2	5	38.4	25.05	13.35	42.6
2	2	5	35.4	23.05	12.35	41.05
2	2	5	36.15	26.45	9.7	39.9
2	2	5	35.5	26.4	9.1	45.9
2	2	5	29.25	20.2	9.05	45.55
2	2	5	37.4	24.4	13	49.45
2	2	5	35.55	26.25	9.3	44.65
2	2	4	34.05	22.6	11.45	39.05
2	2	5	30.2	19.5	10.7	34.25
2	2	4	38.4	25.85	12.55	35.95
2	2	5	37.2	24.85	12.35	42.75
2	2	5	34.15	21	13.15	35.4
2	2	5	32.7	20.8	11.9	35.2
2	2	5	36.2	24.2	12	39.15
2	3	5	31.8	22.15	9.65	42.5
2	2	5	39.5	28.65	10.85	39.35
2	2	5	34.7	24.7	10	40.05
2	2	5	34.85	24.45	10.4	33.6
2	2	5	38.1	26.6	11.5	37.65
2	2	5	34	23.05	10.95	40.95
2	2	5	39.4	26.45	12.95	37.75
2	2	5	34.15	23.15	11	37.8
2	2	5	28.9	19.7	9.2	32.85
2	2	5	33.55	23.95	9.6	37
2	2	5	30.75	19.7	11.05	39.05
2	2	5	33.65	22.25	11.4	36.65
2	2	5	34.65	24.3	10.35	36.05
2	2	5	34.9	23.3	11.6	38.4
2	2	5	33.1	19.6	13.5	45.1
2	2	5	33.45	22.65	10.8	44.5
2	2	5	35.3	23.6	11.7	46.05
2	2	5	33.1	21.9	11.2	45.4
2	2	5	23.2	13.05	10.15	44.25
2	2	5	37.1	15.95	21.15	42.25
2	2	5	34	20.5	13.5	45.3
2	2	5	32.05	18.15	13.9	52.05
2	2	5	31.8	19.75	12.05	47
2	2	5	31.1	20.5	10.6	46.55

2	2	5	33.3	21.45	11.85	47.85
2	2	5	33.05	19.75	13.3	44.7
2	2	5	27.05	16.6	10.45	46.25
2	2	5	32.05	17.7	14.35	48.9
2	2	5	38.7	25.25	13.45	42.2
2	2	5	35.7	18.3	17.4	39.95
2	2	5	31.45	18.9	12.55	42.45
2	2	5	30.65	17.95	12.7	45.9
2	2	5	32.8	22.75	10.05	45.5
2	2	5	34.3	21.9	12.4	47.65
2	2	5	35.25	21.15	14.1	41.7
2	2	5	32.6	25.65	6.95	39.5
2	2	5	35.6	20.95	14.65	46.45
2	2	5	28.8	15.85	12.95	45.3
2	2	5	36.1	25.05	11.05	44.2
2	2	5	32.8	23.1	9.7	51.65
2	2	5	41	28.6	12.4	47.2
2	2	5	35.35	22.95	12.4	50.45
2	2	4	29.95	19.15	10.8	49.15
2	2	5	36.1	22.7	13.4	50.85
2	2	5	30.9	20.45	10.45	53.6
2	2	5	34	21.1	12.9	43.9
2	2	6	38.5	23.9	14.6	53.1
2	2	5	33.6	22.15	11.45	56.8
2	2	5	38.35	23.65	14.7	49.4
2	2	5	38.35	24.3	14.05	41.5
2	2	5	38.7	22.8	15.9	48.7
2	2	5	43.35	28.15	15.2	49.9
2	2	5	40.3	26.5	13.8	46.15
2	2	5	37.95	28.65	9.3	50.1
2	2	5	44.9	31.7	13.2	43.55
2	2	5	41.1	27.75	13.35	57.95
2	2	5	37.55	21.1	16.45	44.35
2	2	5	42.2	30.55	11.65	46.95
2	2	5	29.8	20.15	9.65	42.05
2	2	5	37.1	23.25	13.85	47.2
2	2	5	41.05	30.25	10.8	44.65
2	2	5	38.1	23.45	14.65	43.7
2	2	5	43.4	27.05	16.35	48.55
2	2	5	39.7	27.65	12.05	47.05
2	2	5	41	28.3	12.7	46.65
2	2	5	42	28.7	13.3	53.65
2	2	5	42.8	30.25	12.55	45.6
2	2	5	42.45	28.6	13.85	45.3
2	2	5	39.5	28.05	11.45	40.7
2	2	5	35	22.5	12.5	45.2
2	2	5	44.1	31.1	13	46.05

2	2	5	32.35	19	13.35	36
2	2	5	35.6	21.25	14.35	37
2	2	5	42.85	27.45	15.4	49.1
1	2	6	46.6	34.7	11.9	63.55
1	2	5	47.95	37.55	10.4	55.25
1	2	5	44.2	34.95	9.25	59.85
1	2	6	34.7	20.7	14	59.95
1	2	5	42.55	31.5	11.05	61.95
1	2	5	44.3	34.05	10.25	58.15
1	2	6	40.65	28.1	12.55	55.9
1	2	5	40.6	30.6	10	52.3
1	2	5	43.05	30.15	12.9	59.55
1	2	5	47.05	35.1	11.95	65.15
1	2	5	47	32.25	14.75	63.5
1	2	5	43.05	32.95	10.1	64.95
1	2	5	47.75	35.75	12	47.95
1	2	5	45.4	31.5	13.9	59.35
1	2	6	36.5	22.4	14.1	61.95
1	2	5	44.4	32.45	11.95	53.25
1	2	5	48.7	37.7	11	54.95
1	2	5	47.2	30.15	17.05	53.65
1	2	5	45.95	33.65	12.3	62
1	2	5	46	31.65	14.35	59.1
1	2	5	43.25	31.4	11.85	51.3
1	2	5	42.3	31.4	10.9	56.25
1	2	5	44.95	30.2	14.75	54.9
1	2	5	40.95	26.7	14.25	54.65
1	2	5	35.9	25.3	10.6	34.95
1	2	4	39.7	27.95	11.75	44.35
1	2	5	39.8	27.9	11.9	48.1
1	2	5	41.5	28.95	12.55	53.45
1	2	5	41.7	30.2	11.5	53.15
1	2	5	41.4	26.6	14.8	55.05
1	2	5	41.15	30.65	10.5	52.25
1	2	5	43.2	30.3	12.9	50.7
1	2	5	45.95	33.05	12.9	48.95
1	2	5	39.3	29.15	10.15	48.05
1	2	5	35.3	22.85	12.45	52.2
1	2	5	40.95	29.05	11.9	49.9
1	2	5	34.65	23.15	11.5	47.55
1	2	5	37.05	26.85	10.2	45.4
1	2	5	43.35	32.35	11	43.5
1	2	6	37.8	29.65	8.15	51
1	2	5	33.4	21.2	12.2	37.2
1	2	5	35.45	21.8	13.65	36.9
1	2	4	35.05	23.1	11.95	36.05
1	2	5	35.55	22.85	12.7	35.4

1	2	5	34.9	22.7	12.2	37.8
1	2	5	36.2	23.75	12.45	41
1	2	5	34.25	20.9	13.35	42.9
1	2	5	38.45	22.75	15.7	43.4
1	2	5	39.1	27.2	11.9	46.6
1	2	5	31	17.6	13.4	34.6
1	2	5	35.6	21.7	13.9	40.95
1	2	5	30.65	17.85	12.8	47.9
1	2	5	27.2	14.95	12.25	37.1
1	2	5	28.35	18.55	9.8	45.4
1	2	5	29.25	18.3	10.95	32.7
1	2	5	31.7	20.35	11.35	39.6
1	2	5	36.3	22.8	13.5	44.9
1	2	5	34.35	21.4	12.95	40.1
1	2	5	35.15	23.1	12.05	41.1
1	2	5	31.7	20.35	11.35	39.5
2	2	5	39	29.65	9.35	50.15
2	2	5	47.1	34.55	12.55	48.2
2	2	5	45.05	33.9	11.15	44.05
2	2	5	45.3	30.1	15.2	49.3
2	2	6	48.7	37.55	11.15	47.8
2	2	5	44.45	31.65	12.8	57.05
2	2	5	44	33.45	10.55	51.2
2	2	5	42.05	32.35	9.7	53
2	2	5	44.8	32.1	12.7	54.4
2	2	5	40.55	27.55	13	48.3
2	2	5	39.5	32.15	7.35	49.05
2	2	5	42.65	30.1	12.55	48.7
2	2	5	41.05	27.6	13.45	50.3
2	2	5	40.45	30.3	10.15	47.3
2	2	5	43.35	32	11.35	47.95
2	2	5	46.1	33.4	12.7	51.5
2	2	5	44.3	33.1	11.2	47.55
2	2	5	45.1	31.3	13.8	52
2	2	5	47.15	32.3	14.85	45.95
2	2	5	43.2	37.2	6	53.35
2	2	5	44.9	36.6	8.3	43
2	2	5	33.3	22.9	10.4	35.05
2	2	5	33.95	26.05	7.9	41.9
2	2	5	38.3	26.1	12.2	40.95
2	2	5	41.4	26.6	14.8	31.3
2	2	5	39.55	31.2	8.35	43.15
2	2	5	36.7	25.2	11.5	45.25
2	2	5	34.8	24.4	10.4	36
2	2	4	33.7	27.6	6.1	40.3
2	2	5	40.5	28.3	12.2	52.65
2	2	6	44.75	26.45	18.3	56.1

2	2	5	37.8	25.65	12.15	40.15
2	2	5	40.15	24.9	15.25	40.45
2	2	5	38.45	25.05	13.4	43.5
2	2	5	33.1	21.4	11.7	37.45
2	2	5	31.1	16.95	14.15	38.9
2	2	5	34.95	26.3	8.65	39.05
2	2	5	37	25.6	11.4	41.2
2	2	5	36.55	24.7	11.85	34.85
2	2	5	42	26.55	15.45	43.3
2	2	6	32	21.5	10.5	41.1
2	2	5	38.6	26.6	12	39.5
2	2	5	35.65	25.2	10.45	46.2
2	2	5	33.7	22.4	11.3	38.25
2	2	5	35.8	26.3	9.5	38.95
2	2	5	36.95	27.55	9.4	44.1
2	2	5	38	26.5	11.5	38.7
2	2	5	33.95	23.8	10.15	41.5
2	2	6	33.35	25.8	7.55	44
2	2	6	39.4	28.75	10.65	48.9
2	2	5	35.05	24.3	10.75	40.25
2	2	5	36.9	26.1	10.8	47.1
2	2	5	37.85	28.55	9.3	34.8
2	2	5	35.85	26.2	9.65	34.9
2	2	6	32.5	21.25	11.25	45
2	2	5	39.1	26.6	12.5	40.25
2	2	5	36.6	24.7	11.9	34.55
2	2	5	38.05	27.4	10.65	43.15
2	2	5	38.15	27.8	10.35	42.3
2	2	5	37.9	27.6	10.3	44.25
1	2	5	22.7	15.05	7.65	44
1	2	5	23.4	16.7	6.7	50.35
1	2	5	17.45	10.4	7.05	54
1	2	5	25.1	14.7	10.4	49.85
1	2	5	26.25	18.55	7.7	48.35
1	2	5	23.5	11.95	11.55	48.9
1	2	5	23.8	13.7	10.1	45.8
1	2	5	25.6	17.15	8.45	49.9
1	2	5	23.3	14.95	8.35	44.95
1	2	5	21.75	14.8	6.95	49
1	2	5	20.5	14.55	5.95	55.7
1	2	5	30.35	19.9	10.45	56.85
1	2	6	23.7	14.9	8.8	43.25
1	2	5	29	22.4	6.6	49.3
1	2	5	25	17	8	50.15
1	2	5	34.75	18.55	16.2	52.55
1	2	5	24.95	10.15	14.8	49.9
1	2	4	32.5	18.75	13.75	52.25

1	2	5	29.3	20.2	9.1	55.65
1	2	5	30.9	21.25	9.65	51.2
1	2	5	28.55	16.6	11.95	44.25
1	2	5	36.1	25	11.1	49.95
1	2	5	32.6	21.6	11	46.95
1	2	5	31.2	22.1	9.1	47.5
1	2	5	34	23.1	10.9	48.55
1	2	6	33.35	21.95	11.4	51.6
1	2	5	32	22.2	9.8	46
1	2	5	35.1	23.2	11.9	45.6
1	2	5	32.9	24.2	8.7	47.35
1	2	5	28.3	17.1	11.2	45.9
1	2	5	30.05	17.9	12.15	46.35
1	2	5	29.05	17.95	11.1	47.3
1	2	5	30.05	19.05	11	48
1	2	5	33	21.65	11.35	46.6
1	2	5	30.5	19.95	10.55	44.8
1	2	5	41	29.05	11.95	45.65
1	2	5	34.45	22.25	12.2	44.9
1	2	5	28.3	19.25	9.05	49.95
1	2	5	30.25	19.3	10.95	49.3
1	2	5	41.05	28.85	12.2	46.1
2	2	5	34.15	20.85	13.3	36.1
2	2	5	32.9	20.35	12.55	36.9
2	2	5	36.05	22.8	13.25	33.4
2	2	5	37	24.7	12.3	37.3
2	2	6	44	32.95	11.05	56
2	2	7	42.9	32.4	10.5	50.8
2	2	5	34.15	23.05	11.1	38.3
2	2	5	36.4	23.4	13	35.5
2	2	5	45.7	31.6	14.1	49.25
2	2	6	30.45	22.5	7.95	40.3
2	2	5	31.6	20.95	10.65	37.3
2	2	6	33.3	23.55	9.75	36.35
2	2	6	28.95	18	10.95	37.9
2	2	6	31.9	18.85	13.05	34.2
2	2	5	34.6	24.4	10.2	32.65
2	2	5	31.55	20.4	11.15	33.2
2	2	6	29.4	20.35	9.05	32.2
2	2	5	32.6	21.75	10.85	28.8
2	2	5	28.65	18.1	10.55	28.3
2	2	5	32.9	21.5	11.4	29.05
2	2	5	32.4	24.25	8.15	41.3
2	2	4	36.55	26.35	10.2	44.9
2	2	5	25.5	17.25	8.25	47
2	2	5	32.5	26.25	6.25	44
2	2	5	33.25	26.05	7.2	47.7

2	2	5	27.95	17.3	10.65	48.55
2	2	5	31.6	24	7.6	46.3
2	2	5	30.85	22.35	8.5	45.4
2	2	5	22.45	14.1	8.35	47
2	2	5	35	25.7	9.3	43.05
2	2	5	20.1	10.9	9.2	43.05
2	2	4	25.95	16.9	9.05	40.4
2	2	5	27.35	18.6	8.75	40.95
2	2	4	24.05	14.65	9.4	32.95
2	2	5	22.85	12.9	9.95	41.7
2	2	5	30.8	19.75	11.05	43.7
2	2	5	33.15	24.85	8.3	47
2	2	6	38.5	16.8	21.7	41.2
2	2	5	26.1	18.7	7.4	45.4
2	2	5	24.15	13.25	10.9	39.8
2	2	5	39.9	26.35	13.55	46.65
2	2	5	38.9	27.45	11.45	53.95
2	2	5	30.6	17.8	12.8	52.7
2	3	5	36.6	23.7	12.9	52
2	3	6	36.35	25.45	10.9	54.7
2	2	5	38.75	25.3	13.45	47.8
2	2	5	39.6	23.85	15.75	49.8
2	2	5	41.4	27.4	14	43.65
2	2	5	41.5	27.75	13.75	46.55
2	2	5	37.45	25.15	12.3	51.45
2	2	5	39.5	29.65	9.85	49
2	2	4	40.3	26.65	13.65	44.3
2	2	5	35	22.8	12.2	48.1
2	2	5	35.6	29.05	6.55	52.05
2	2	5	41.4	27.05	14.35	47.2
2	2	6	39.35	25.8	13.55	50.6
2	2	6	35.4	22.45	12.95	49.1
2	3	5	39.25	26.1	13.15	52.5
2	2	5	40.6	24.65	15.95	53.1
2	3	6	35.9	24.45	11.45	55.8
1	2	4	20.55	13.6	6.95	43.1
1	2	5	27.75	18.9	8.85	47.45
1	2	5	34.35	25.4	8.95	41.6
1	2	5	32.3	23.7	8.6	46.5
1	2	5	41.3	32.9	8.4	45.1
1	2	4	26.85	17.2	9.65	41.6
1	2	5	30	21.3	8.7	54.6
1	2	5	25.45	17.8	7.65	49.9
1	2	5	37.25	28	9.25	46.1
1	2	5	31.25	24.3	6.95	46.7
1	2	4	26.5	18.35	8.15	46.8
1	2	5	39.2	28.1	11.1	46.95

1	2	5	20.35	13.75	6.6	48.55
1	2	5	28.35	18.85	9.5	43.6
1	2	4	27.05	17.65	9.4	45.1
1	2	5	42.6	31.2	11.4	50.45
1	2	4	30.8	22.65	8.15	40.3
1	2	5	41.9	30.65	11.25	39.95
1	2	5	32.1	21.15	10.95	51
1	2	5	19.25	12.15	7.1	40.5
1	2	5	37.85	30.25	7.6	47
1	2	5	39.85	24.25	15.6	44.65
1	2	5	28.25	18.3	9.95	41.7
1	2	5	33.5	25.45	8.05	50.35
1	2	5	40.1	28.4	11.7	45.95
1	2	5	33.5	23.55	9.95	53.2
1	2	5	37.05	25.5	11.55	56.2
1	2	5	35.85	21.6	14.25	47.3
1	2	5	38.25	23.1	15.15	52.95
1	2	5	36.85	23.4	13.45	48.55
1	2	5	39.55	28.6	10.95	50.25
1	2	5	35.9	24.45	11.45	46.6
1	2	5	42.45	32.95	9.5	48.55
1	2	5	39.9	29.1	10.8	50.75
1	2	5	40.4	31.85	8.55	52.25
1	2	5	40.8	24.6	16.2	52.4
1	2	5	36.45	20.65	15.8	53.2
1	2	5	38.55	29.65	8.9	51.35
1	2	5	37.2	24.4	12.8	52.95
1	2	5	34.8	24.95	9.85	56.7
1	2	5	43	22.25	20.75	43.25
1	2	5	44.1	33.8	10.3	48.6
1	2	5	41.55	31.25	10.3	46.6
1	2	5	39.6	29	10.6	36.5
1	2	5	40.9	30.45	10.45	41.6
1	2	5	40.85	28.9	11.95	46.3
1	2	5	39.15	25.85	13.3	49.7
1	2	5	43.05	32.2	10.85	53.15
1	2	5	39.8	26.2	13.6	53.95
1	2	5	46.6	34.8	11.8	46.4
1	2	5	34.8	22.3	12.5	30.65
1	2	5	40.1	29	11.1	51
1	2	5	35.55	27.2	8.35	40.3
1	2	5	34.2	22.5	11.7	39.05
1	2	5	44.45	34	10.45	31.5
1	2	5	41.4	31.25	10.15	42.2
1	2	4	42.75	32.2	10.55	45.05
1	2	5	41.7	28.5	13.2	54.6
1	2	5	42.5	33	9.5	47.15

1	2	5	47	32.1	14.9	56.95
2	2	5	44.7	29.25	15.45	58.45
2	2	5	39.4	24.8	14.6	56.35
2	2	5	37.4	28.2	9.2	50.1
2	2	5	41.4	25.1	16.3	53.5
2	2	5	36.8	21.6	15.2	52.05
2	2	5	43.05	27.55	15.5	47.2
2	2	5	36.8	23.95	12.85	56.35
2	2	5	34.9	24.6	10.3	56
2	2	5	34.45	25.7	8.75	53.8
2	2	6	41.6	27.7	13.9	51.1
2	2	5	32.05	20.9	11.15	49.75
2	2	5	38.2	19.8	18.4	55.5
2	2	5	33.8	21.1	12.7	57.4
2	2	5	31.9	17.45	14.45	51
2	2	5	27.45	14.45	13	48.05
2	2	5	31.6	16.5	15.1	58.4
2	2	5	26.85	12	14.85	50.55
2	2	5	35.7	24.9	10.8	61.45
2	2	5	39.55	25.45	14.1	46.95
2	2	5	28.3	16.4	11.9	53.95
2	2	5	29.6	17.8	11.8	43.3
2	2	5	37.4	25.85	11.55	51.9
2	2	5	37.3	27	10.3	53.55
2	2	5	38.9	17.4	21.5	32.35
2	2	5	22.9	18.2	4.7	35.1
2	2	5	25.8	15.85	9.95	29.3
2	2	5	36.2	24.1	12.1	33.25
2	2	5	31.05	20.6	10.45	34.15
2	2	5	22.25	13.2	9.05	35.3
2	2	5	31.9	19.7	12.2	34.5
2	2	5	26.5	14.15	12.35	36.65
2	2	5	31.9	16.75	15.15	36.45
2	2	5	30.55	16.65	13.9	23.3
2	2	5	29.2	17.2	12	33.05
2	2	5	37.4	25.85	11.55	55.05
2	2	5	36.6	23.1	13.5	46.5
2	2	5	40.7	26.8	13.9	41.6
2	2	5	38.8	25.25	13.55	43.8
2	2	5	38.8	24.4	14.4	51.85
2	2	5	44.2	31.05	13.15	53.2
2	2	5	36.1	23.75	12.35	42.7
2	2	5	40.8	26.95	13.85	44.25
2	2	5	39.8	24.5	15.3	45.95
2	2	5	36.3	21.9	14.4	49.15
2	2	5	34.3	25.2	9.1	41.15
2	2	5	35	21.6	13.4	47.25

2	2	5	35.2	22.2	13	49.05
2	2	5	37.85	23.85	14	49
2	2	5	32.5	18	14.5	47.3
2	2	5	37.9	23.9	14	44.85
2	2	5	36.05	20.75	15.3	52.8
2	2	6	35.15	22.8	12.35	50.95
2	2	5	36.8	23.7	13.1	47.7
2	2	5	37.7	25.8	11.9	46.25
2	2	5	38.6	23.95	14.65	47.4
2	2	5	36.3	23.45	12.85	44.8
2	2	5	30.2	21.85	8.35	51.05
2	2	5	37.65	27.95	9.7	36.9
2	2	5	38.3	23	15.3	50.5
2	2	5	40.3	27.8	12.5	43.1
2	2	5	31.7	22.25	9.45	52.2
2	2	5	36.2	24.75	11.45	51.4
2	2	5	29.15	16.3	12.85	49.95
2	2	5	30.7	18.2	12.5	36.8
2	2	5	30.55	18.9	11.65	46.2
2	2	5	27.55	14	13.55	42.1
2	2	5	30.25	19.95	10.3	45.5
2	2	4	35.65	23.65	12	34.65
2	2	5	30.4	21.6	8.8	43.45
2	2	5	31.2	18.3	12.9	41.7
2	2	6	32.35	19.9	12.45	57.95
2	2	5	37.95	23.9	14.05	44.85
2	2	5	36.1	21.4	14.7	42.4
1	2	5	34.7	23.8	10.9	46.05
1	2	5	33.4	22.4	11	47
1	2	6	31.45	20.2	11.25	30.2
1	2	6	31.4	20.2	11.2	30.2
1	2	5	30.5	20.3	10.2	46.15
1	2	5	28.1	17.1	11	29.25
1	2	5	33	20.4	12.6	36.2
1	2	5	30	18.05	11.95	35.5
1	2	5	36.3	23.15	13.15	39.05
1	2	5	35	22.35	12.65	37.4
1	2	5	33.6	21.2	12.4	39.25
1	2	7	31.8	17.2	14.6	45.95
1	2	6	28.6	14.8	13.8	40.3
1	2	6	34.2	23.95	10.25	42.4
1	2	5	32.6	21.8	10.8	35
1	2	5	39.4	15.55	23.85	40.3
1	2	5	30.25	21.9	8.35	32
1	2	6	30.05	17.9	12.15	41.9
1	2	5	32.2	21.4	10.8	32.25
1	2	6	31.35	22.05	9.3	42

1	2	5	45.4	31.3	14.1	50.85
2	2	5	26.2	18.1	8.1	38.9
2	2	5	23.35	14.8	8.55	43
2	2	5	26.85	14.45	12.4	32.05
2	2	5	24.25	15.65	8.6	43.05
2	2	5	32.1	22.4	9.7	40.35
2	2	5	35.4	23.35	12.05	42.45
2	2	5	25.55	19.9	5.65	35.35
2	2	5	27.95	19.35	8.6	44.35
2	2	5	21.9	12.75	9.15	34.75
2	2	5	23.4	16.25	7.15	39
2	2	5	34.2	25.25	8.95	51.9
2	2	5	34.5	28.35	6.15	50.4
2	2	5	35.45	22.75	12.7	51.8
2	2	5	34.5	23.45	11.05	42.3
2	2	5	32	23.7	8.3	46.95
2	2	4	36.05	26.35	9.7	40.2
2	2	5	22.75	15.45	7.3	40.05
2	2	5	29.65	20.05	9.6	40
2	2	5	23.8	15.9	7.9	38.75
2	2	5	26.1	16.5	9.6	41.5
2	2	5	33.7	20.8	12.9	50.05
2	2	5	41.25	29.8	11.45	49.7
2	2	5	43.95	32.1	11.85	39.8
2	2	5	38.95	23.4	15.55	49
2	2	5	46.5	34.05	12.45	44.3
2	2	5	33.85	23.45	10.4	36.15
2	2	5	40.25	25.95	14.3	26.7
2	2	5	38.95	26.85	12.1	25.4
2	2	5	40	22.15	17.85	53.6
2	2	6	32.35	22.6	9.75	53.15
2	2	5	39.35	27.8	11.55	48.05
2	2	6	31.1	21.1	10	40.85
2	2	6	27.45	15.85	11.6	51.85
2	2	5	36.55	28.05	8.5	51.4
2	2	5	33.95	22.3	11.65	42.25
2	2	5	31.15	21.8	9.35	51.6
2	2	5	35.25	19.25	16	44.95
2	2	5	36.95	26.2	10.75	44
2	2	5	31.6	21.7	9.9	46.85
2	2	5	36.4	23.65	12.75	42
2	2	5	38.9	27.25	11.65	41.6
2	2	5	29.95	19.3	10.65	43.45
2	2	5	32	18.75	13.25	48.2
2	2	6	26.5	16	10.5	42.15
2	2	6	26.4	14.9	11.5	43.3
2	2	5	25.25	13.2	12.05	38.7

2	2	6	30.6	18.75	11.85	42.25
2	2	5	32.4	25.55	6.85	45.4
1	2	5	33.95	21.5	12.45	54.7
1	2	5	39.5	22.95	16.55	55.25
1	2	5	40.7	25.65	15.05	55.7
1	2	5	36.6	23.4	13.2	50.4
1	2	5	34.15	20.9	13.25	55.95
1	2	6	41.65	29.8	11.85	62.75
1	2	5	38.8	29	9.8	57.1
1	2	5	39.85	31.8	8.05	50.65
1	2	5	41.3	24.25	17.05	56.5
1	2	5	40.1	25.6	14.5	63.95
1	2	4	41.85	29.4	12.45	49.2
1	2	5	32.3	22.1	10.2	58.6
1	2	5	35.45	23.8	11.65	50.25
1	2	3	30.95	22.1	8.85	31.05
1	2	5	27.75	22.3	5.45	43
1	2	6	32.5	24.3	8.2	54.35
1	2	5	38.5	25.25	13.25	27.55
1	2	5	38.3	28.5	9.8	30.8
2	2	5	19.55	10.35	9.2	37.35
2	2	5	21.2	12.2	9	32.65
2	2	5	24.7	6.45	18.25	39.4
2	2	5	20.5	10.85	9.65	32.15
2	2	5	26.4	17.5	8.9	40.1
2	2	5	19.65	8.25	11.4	37.4
2	3	5	25.9	18.35	7.55	41.6
2	2	5	23.7	15.3	8.4	35.25
2	2	5	23.2	15.4	7.8	33.35
2	2	5	24.75	14.5	10.25	32.25
2	2	5	29.75	20.3	9.45	37.8
2	2	5	20.5	11.95	8.55	30.5
2	2	5	17.25	10.25	7	34.9
2	2	5	15.6	8.3	7.3	30.4
2	2	6	21.55	13.1	8.45	38.4
2	2	5	15.45	8.9	6.55	32.85
2	2	5	21.65	15.1	6.55	41.05
2	2	5	24.55	15.2	9.35	37.55
2	2	5	23.85	17.15	6.7	35.7
2	2	5	22.45	9	13.45	31.75
1	2	5	32.7	19.8	12.9	51.4
1	2	5	29.15	13.4	15.75	43.95
1	2	5	24.55	11.95	12.6	42.6
1	2	5	29.7	18.5	11.2	40.8
1	2	5	30	17.2	12.8	42.7
1	2	5	32.25	19.5	12.75	47.15
1	2	5	26.2	12.3	13.9	40.95

1	2	5	31.5	19.9	11.6	46.05
1	2	5	31.1	17.95	13.15	46.35
1	2	5	27.6	14.95	12.65	47.6
1	2	5	28.05	16.4	11.65	45
1	2	5	27.7	15.85	11.85	44.15
1	2	5	26.4	15.1	11.3	40
1	2	5	27.5	19.2	8.3	20.1
1	2	5	25.8	12.25	13.55	42.3
1	2	5	22.9	11.5	11.4	44.2
1	2	5	34.55	20.7	13.85	45.2
1	2	5	29.4	19.7	9.7	48.15
1	2	5	32.6	20.45	12.15	44.25
1	2	5	31.7	16.15	15.55	42.2
1	2	5	26.6	12.85	13.75	49.8
1	2	5	30.1	17.75	12.35	53
1	2	5	28	15.9	12.1	47.65
1	2	6	23.25	12.95	10.3	49.3
1	2	5	29.3	16.7	12.6	47.2
1	2	5	34.8	21.05	13.75	46.45
2	2	5	34.7	23.3	11.4	39.1
2	2	5	38.95	26.9	12.05	47.65
2	2	5	34.95	23.15	11.8	37
2	2	5	25.6	18.1	7.5	45.6
1	2	5	19.45	9.7	9.75	35.3
1	2	5	21.9	12.75	9.15	44.6
1	2	5	23.25	13	10.25	47
1	2	5	22.4	11.95	10.45	45
1	2	5	23.3	13.1	10.2	44.5
1	2	6	20.75	11.7	9.05	46.95
1	2	5	25.3	14.85	10.45	48.05
2	2	5	35.5	24.85	10.65	34.9
2	2	5	28.25	22.05	6.2	39.95
2	2	5	34.15	20.95	13.2	31.95
2	2	5	32.5	27.8	4.7	37.75
2	2	5	37.65	27.8	9.85	42.4
2	2	5	35.25	23.35	11.9	34.25
1	2	5	36.6	24.2	12.4	36.7
1	2	5	34.1	22.9	11.2	36
1	2	5	34.95	20.95	14	36.85
1	2	5	41.15	24.05	17.1	35.2
1	2	5	35.95	22.5	13.45	40.9
1	2	5	35.2	23.4	11.8	44.45
1	2	5	40.05	29.5	10.55	39.05
1	2	3	36.5	24.95	11.55	43.45
1	2	5	36.15	25.65	10.5	42.85
1	2	5	39.3	27.65	11.65	40
1	2	5	39.65	26.45	13.2	38.55

2	2	5	38.4	24.85	13.55	41.8
2	2	5	37.25	26.7	10.55	49.2
2	2	5	39.5	29.95	9.55	51.9
2	2	5	27.95	19.25	8.7	34.15
2	2	5	31.65	19.5	12.15	18.6
2	2	5	30.8	18.85	11.95	30.55
2	2	6	26.05	20.4	5.65	27.4
2	2	5	38.15	27.6	10.55	32.95
2	2	5	33.55	24.65	8.9	35.35
2	2	5	35.9	25.25	10.65	33.8
2	2	5	31.6	20.5	11.1	33.65
2	2	5	32.15	22.25	9.9	36.85
2	2	5	32.7	17.85	14.85	36.2
2	2	5	30.6	21.65	8.95	30.4
2	2	5	35	23.8	11.2	36.15
1	2	6	24.85	14.75	10.1	42.5
1	2	5	30.4	23.45	6.95	41.5
1	2	4	33.85	24.55	9.3	39.85
1	2	5	30	20.4	9.6	33.7
1	2	5	29.7	21.85	7.85	38.35
1	2	5	26.2	19.15	7.05	38
1	2	4	25.75	19.9	5.85	31.95
1	2	5	24.45	17.55	6.9	36.5
1	2	6	26	19.55	6.45	37.2
1	2	5	23.85	16.1	7.75	41.7
1	2	7	21.05	11.2	9.85	45.6
1	2	6	22.7	18.1	4.6	32.2
1	2	5	21.85	13.7	8.15	33.5
1	2	4	24.85	15.8	9.05	32.1
1	2	4	27.85	19.2	8.65	30.15
1	2	6	23.45	14.4	9.05	38.35
1	2	5	27	17	10	32.5
1	2	5	27.2	17.95	9.25	35.75
1	2	4	24.2	15.8	8.4	35.35
1	2	5	27	17.5	9.5	39.75
1	2	5	29.45	19.7	9.75	42.95
1	2	5	27.85	17	10.85	46.2
1	2	5	35.2	23.55	11.65	39.8
1	2	5	34.25	23.6	10.65	44.7
1	2	5	32.65	22.15	10.5	42.3
1	2	5	41.95	30.5	11.45	34.2
1	2	5	47.7	36.45	11.25	35.35
1	2	5	39.35	30.25	9.1	29.5
1	2	5	40.05	30.9	9.15	32.4
1	2	5	42.75	32.5	10.25	34.65
1	2	4	30	20.9	9.1	47.7
1	2	5	26.5	18.4	8.1	40.7

1	2	6	25.4	10.15	15.25	40
1	2	5	34	22.05	11.95	36.95
1	2	5	25.9	17.75	8.15	41.7
1	2	5	33.7	24.8	8.9	48.65
1	2	5	34.65	20.5	14.15	50
1	2	5	26.45	17.85	8.6	42.65
1	2	5	31.4	18.6	12.8	42.2
1	1	5	28.25	15.75	12.5	41.15
1	2	5	28.4	20	8.4	48
1	2	5	25.55	16.7	8.85	43.4
1	2	5	36.3	27.35	8.95	42
1	2	5	32.85	22.5	10.35	46.8
1	2	5	25.35	16.8	8.55	44.65
1	2	4	35.8	25.2	10.6	41
1	2	5	34.25	24	10.25	47.75
1	2	5	36.15	25.45	10.7	42.45
1	2	5	32.45	22.9	9.55	42.95
1	2	5	27.35	19.5	7.85	42.95
1	2	5	25.9	12.1	13.8	35.75
1	2	6	21.75	10.35	11.4	32.65
1	2	6	23.85	13.45	10.4	38.2
1	2	5	27.8	20.2	7.6	33.15
1	2	5	25.2	16.4	8.8	32.1
1	2	5	29.2	18.05	11.15	31.6
1	2	5	28.95	20.2	8.75	28.6
1	3	5	26.1	17.5	8.6	30.5
1	2	5	22	10.6	11.4	39.8
1	2	5	22.95	14.65	8.3	31.6
1	2	5	24.6	16.6	8	32.7
1	2	5	29.2	19.55	9.65	33.7
1	2	5	20.4	12.15	8.25	29.7
1	2	5	24.9	16.8	8.1	29.95
1	3	5	24.95	15.3	9.65	32.55
1	2	5	25.65	13.6	12.05	28.85
1	2	5	27.2	16.7	10.5	35
1	2	5	27.35	17.5	9.85	29.4
1	2	5	25.6	15.7	9.9	32.55
1	2	5	25.25	14.6	10.65	35.35
1	2	4	32.25	24.4	7.85	40.6
1	2	5	29.7	19.9	9.8	36.65
1	2	5	36.5	24.7	11.8	39.1
1	3	5	30.25	21.1	9.15	36.2
1	2	5	36.4	25.45	10.95	38.05
1	2	5	37.6	27.9	9.7	43.95
1	3	6	33.5	22.8	10.7	44.35
1	2	5	34.85	22.95	11.9	39.1
1	2	6	32.35	21	11.35	40.75

1	3	5	32.35	26.45	5.9	43.1
1	2	5	25.6	19.9	5.7	41.15
1	2	5	33.7	22.1	11.6	38.05
1	2	5	26.6	17.45	9.15	34.35
1	2	4	27.65	16.2	11.45	34.95
1	2	3	35.4	27.55	7.85	36.7
1	2	4	27.35	20.55	6.8	34.05
1	2	5	29.15	19	10.15	39.2
1	2	5	34.15	25.6	8.55	40.25
1	2	5	32.5	23	9.5	33.75
1	2	5	40.25	30.4	9.85	42.4
1	2	5	34.8	20.05	14.75	42.7
1	2	5	38	24.3	13.7	40.15
1	2	5	33.65	22.8	10.85	43.95
1	2	5	30.35	20.4	9.95	37.5
1	2	5	34.2	21.45	12.75	37.55
1	2	5	33.1	21.1	12	37.6
1	2	5	35.1	23.7	11.4	38.5
1	2	5	32.05	21.3	10.75	35.45
1	2	5	34.95	20	14.95	37.5
1	2	6	33.75	21.7	12.05	34.55
1	3	5	35.45	23.25	12.2	31.55
1	3	5	31.75	17.2	14.55	37.15
1	3	6	35.1	25.75	9.35	36.45
1	2	4	31.45	19.6	11.85	38.1
1	3	5	30.6	18.95	11.65	29.4
1	3	5	32.5	19.75	12.75	32.5
1	3	5	33.2	20.45	12.75	34.1
1	2	6	31.25	19.8	11.45	32.95
1	2	5	32.35	21.1	11.25	35.1
1	2	4	27.2	15.15	12.05	34.05
2	2	5	31.65	18.55	13.1	37.3
2	2	5	34.05	22.8	11.25	46.65
2	2	5	34.05	22.85	11.2	46.65
2	2	5	24.1	17.35	6.75	43.9
2	2	5	26.45	15.05	11.4	45.5
2	2	5	35.95	26.6	9.35	33.35
2	2	5	32	22.8	9.2	46.1
2	2	5	25.8	14.05	11.75	39.25
2	2	5	26.4	15.05	11.35	45.5
2	2	5	30.55	19.95	10.6	49
2	2	5	28.2	14.4	13.8	44.4
2	2	5	32.6	24.1	8.5	47.25
2	2	5	26.1	18.05	8.05	40.25
2	2	5	29	19.8	9.2	43.7
2	2	5	29.3	20.7	8.6	43.7
2	2	9	39	29.8	9.2	49

2	2	5	29.65	18.8	10.85	41
2	2	5	33.3	26	7.3	44.3
2	2	5	25.85	15	10.85	43.95
2	2	6	23.65	13.3	10.35	44.2
2	2	5	27.5	19.4	8.1	32.7
2	2	5	25.65	15.5	10.15	40
2	2	5	28.15	13.65	14.5	41.8
2	2	5	32.8	17.1	15.7	34.85
2	2	5	37.4	25.8	11.6	44.95
2	2	5	32.45	20.3	12.15	40
2	2	5	33.55	22.5	11.05	38.45
2	2	5	34.6	20.15	14.45	40

NSCTARGUIDE	LOBE	COLOR
12.5	6	1
13.75	5	1
14.1	6	1
12.8	5	1
12.4	5	1
12.15	5	1
11	6	1
11.95	5	1
12	5	1
12.8	5	1
11.05	5	1
12	5	1
11.55	6	1
10.8	5	1
12.2	5	1
10.1	5	1
10.5	5	1
9.5	5	1
15.8	6	1
10.9	5	1
10.5	4	1
12.25	5	1
12.75	5	1
10.8	5	1
11.75	5	1
11.3	5	1
11.35	5	1
11.3	5	1
10.45	5	1
11.35	6	1
11.4	5	1
12.4	5	1
12.4	6	1
12	5	1
11.35	4	1
10.2	4	1
11.65	5	1
12.45	5	1
11	6	1
11	5	1
12.8	5	1
13.15	5	1
12.45	5	1
13.3	5	1
13.5	5	1
11.6	5	1

13.05	5	1
13.8	5	1
13.2	5	1
13.7	5	1
12.95	5	1
12.55	5	1
12.05	5	1
12.6	5	1
12.55	5	1
11.7	5	1
12.5	5	1
13.05	5	1
12.7	5	1
11.9	5	1
11.8	5	2
13.05	5	2
11.55	5	2
11.45	5	2
12.25	5	2
11.6	5	2
12.9	5	2
11.8	5	2
11.45	6	2
13.75	5	2
13.7	5	2
10.2	5	2
11.5	4	2
12.8	5	2
11.9	5	2
11.7	5	2
12.65	5	2
11.9	5	2
10.65	4	2
13.05	5	2
9.75	5	2
9.8	5	2
11	5	2
11.35	5	2
10.25	5	2
10.95	5	2
9.5	5	2
10.5	5	2
10.6	6	2
10.7	5	2
9.45	5	2
11	5	2
10.8	5	2

10.55	5	2
9.8	5	2
11.15	5	2
10	5	2
9	5	2
12.7	5	2
9.85	4	2
15	5	2
14.75	5	2
12.95	5	2
13	5	2
11.7	5	2
14.9	5	2
12.55	5	2
10.35	5	2
13.45	5	2
11.6	5	2
11.95	5	2
11.95	5	2
11.9	5	2
13.1	5	2
14.6	5	2
12.1	5	2
11.35	5	2
11.65	5	2
12.5	5	2
13.9	5	2
12	5	1
12.1	5	1
11.45	6	1
12.95	5	1
12.45	5	1
12.85	5	1
13.45	5	1
11.35	5	1
11	5	1
13.7	5	1
12.75	5	1
12.2	5	1
10.75	5	1
11.7	5	1
9.65	4	1
13.65	6	1
9.85	4	1
10	5	1
12.7	5	1
10.9	5	1

10.4	5	1
10.5	5	1
11.9	5	1
9.8	5	1
10	5	1
11	5	1
10.8	5	1
11.75	5	1
11.55	5	1
11.1	5	1
12	5	1
9.5	5	1
9.5	5	1
10.55	5	1
10.1	5	1
12.05	5	1
9.55	4	1
9.4	5	1
8.4	4	1
10	5	1
11.25	5	1
11.8	5	1
9.4	5	1
10.65	5	1
10.9	5	1
9.95	5	1
10.45	5	1
12.25	5	1
11.6	5	1
10.8	5	1
10.95	5	1
10.35	5	1
14.65	5	1
13.95	5	1
13.05	5	1
13.3	5	1
12.8	5	1
12.05	5	1
10.6	5	1
11.45	5	1
11.85	5	2
14.3	7	2
10.8	5	2
11.45	6	2
12.55	5	2
11.85	6	2
9.05	5	2

10.15	6	2
11.05	6	2
11.6	6	2
10.8	5	2
11	5	2
11.5	5	2
12.05	6	2
11.65	6	2
10	5	2
11.55	6	2
10.5	5	2
10.75	6	2
10.9	5	2
10.55	5	2
8.7	5	2
11.15	5	2
10.65	5	2
10	5	2
11.25	5	2
10.7	5	2
10.1	5	2
10.35	5	2
10.7	5	2
11.15	5	2
11.5	7	2
9.1	5	2
10.4	5	2
10.3	5	2
11.35	5	2
11.35	5	2
11.2	6	2
10.1	5	2
10	5	2
10.9	5	2
9.6	5	2
11.15	5	2
9.3	5	2
10	5	2
11.9	5	2
12.7	6	2
14.65	6	2
11.25	5	2
11.6	5	2
10.25	5	2
11.2	5	2
11.2	5	2
13.1	7	2

12.95	6	2
10.75	5	2
11.05	5	2
11.5	6	2
11.3	5	2
10.05	6	2
12.5	5	4
14.8	5	4
14.65	5	4
14.6	5	4
10.65	5	4
10.8	5	4
10.65	5	4
14.5	5	4
13.65	5	4
13.4	5	4
12.65	6	4
12.95	5	4
14.2	5	4
12.75	5	4
11.1	5	4
12.8	5	4
12.85	5	4
13.25	5	4
11.75	5	4
11.05	5	4
11.7	5	4
13.65	4	4
10.9	6	4
10.8	5	4
11.15	5	4
12.2	5	4
10.9	5	4
10.55	5	4
10.5	5	4
11.9	5	4
11.25	6	4
11.2	5	4
10.95	5	4
9.95	5	4
11.05	5	4
11.3	5	4
12.25	5	4
11.05	5	4
10.35	5	4
11.35	5	4
11.05	6	4

11.1	5	4
10.35	5	4
11.3	5	4
12.45	5	4
10.9	5	4
11.25	5	4
12.1	5	4
11.4	5	4
10	5	4
11.25	5	4
13.55	5	4
13.9	5	4
10.8	5	4
10.55	6	4
9.8	5	4
11.15	5	4
10	5	4
13	5	4
11.35	5	4
14	5	2
13.65	5	2
14.85	5	2
14.05	5	2
12.7	5	2
14.8	5	2
14.1	5	2
15	5	2
14.9	5	2
14.45	5	2
12.95	5	2
13.1	5	2
13.8	5	2
14.75	5	2
13.5	5	2
14	5	2
13.8	5	2
13.05	5	2
12.1	5	2
15.15	5	2
11.8	5	2
12.45	5	2
11.35	5	2
7.1	4	2
11.55	5	2
10.75	5	2
10.75	5	2
12.5	5	2

11.7	5	2
11.4	5	2
11.85	5	2
12.35	5	2
11.55	5	2
11.2	5	2
13.25	5	2
12.4	5	2
12.95	5	2
14.45	5	2
12.1	5	2
11.05	5	2
17.8	5	2
14.6	5	2
13.65	5	2
17.2	7	2
16.3	5	2
15.7	5	2
18.6	6	2
15.05	5	2
15.7	5	2
13.7	5	2
14.1	5	2
13.85	5	2
15.15	5	2
17.2	6	2
16.7	5	2
15	5	2
14.95	5	2
14.65	8	2
18.45	7	2
15.9	6	2
11.85	5	2
12.35	5	2
12.5	5	2
11.95	5	2
11.9	5	2
12.35	5	2
12.7	5	2
12	5	2
11.25	4	2
13.85	5	2
13.8	5	2
12.75	5	2
14.25	5	2
13.4	6	2
12.2	5	2

15	6	2
12	5	2
13.3	6	2
11.25	5	2
14.35	5	2
8.25	4	2
10.05	5	2
10.8	5	2
9.1	5	2
8.85	5	2
10.45	5	2
10	5	2
10.5	5	2
10.6	5	2
9.05	4	2
8.75	4	2
11.55	5	2
9.7	5	2
10.1	4	2
11.6	5	2
12.6	5	2
11.2	5	2
9.8	5	2
10.25	5	2
11.05	5	2
13.85	5	2
13.8	5	2
12.75	5	2
14.25	5	2
13.4	6	2
12.2	5	2
15	6	2
12	5	2
13.3	6	2
11.25	5	2
14.35	5	2
8.25	4	2
10.05	5	2
10.8	5	2
9.1	5	2
8.85	5	2
10.45	5	2
10	5	2
10.5	5	2
10.6	5	2
11.75	5	1
10.05	5	1

11.6	5	1
10.7	5	1
10.9	5	1
11.4	5	1
10.7	5	1
10.9	5	1
10.5	5	1
12.5	6	1
11.1	5	1
13.6	5	1
11.8	5	1
11.05	5	1
10.2	5	1
10.2	5	1
11.2	5	1
10.85	5	1
10.75	5	1
12.25	5	1
8.65	5	1
8.45	5	1
10.45	5	1
10.3	5	1
9.35	5	1
9.9	5	1
10.8	5	1
8.65	5	1
9	5	1
9.6	5	1
9.75	5	1
9.25	5	1
10.05	5	1
10.55	5	1
11.05	5	1
10.1	5	1
9.85	5	1
9.1	5	1
9.7	5	1
10.4	5	1
12.65	5	1
13.15	6	1
13.05	5	1
12.3	5	1
12.15	6	1
12.25	7	1
14.15	6	1
11.55	6	1
12.05	5	1

10.5	5	1
12.85	5	1
11.85	5	1
12.6	5	1
13.05	7	1
12.85	6	1
12.95	5	1
12.25	5	1
12.3	7	1
11.9	6	1
10.95	5	1
11.7	5	2
11.2	5	2
12.75	5	2
12.95	5	2
12.75	5	2
13.2	5	2
11	5	2
11.55	5	2
10.95	5	2
12.6	5	2
11.05	4	2
11.2	5	2
10.8	4	2
13	5	2
13.05	5	2
11.3	5	2
12.65	5	2
12.35	5	2
12.45	5	2
12	5	2
10.3	5	2
10.5	5	2
10	5	2
10.65	4	2
10	6	2
9.55	5	2
9.75	4	2
9.75	5	2
8.85	5	2
10.65	5	2
9.2	5	2
8.2	5	2
10.7	5	2
10.65	5	2
10	5	2
10.85	5	2

8.7	5	2
9	4	2
8.4	5	2
8.05	5	2
11.05	5	2
11.2	4	2
10.8	5	2
13	4	2
13.05	5	2
11.3	5	2
12.65	5	2
12.35	5	2
12.45	5	2
12	5	2
10.3	5	2
10.5	5	2
10	5	2
10.65	5	2
10	4	2
9.55	6	2
9.75	5	2
9.75	4	2
8.85	5	2
10.65	5	2
8.35	4	2
10	4	2
10.3	5	2
9.65	5	2
10.75	5	2
9.65	5	2
10.75	5	2
11.55	5	2
10	6	2
8.65	5	2
8.55	4	2
11	6	2
10.7	5	2
9	5	2
10.75	5	2
12.55	5	2
12	5	2
9.4	5	2
9.5	5	2
12	6	2
13.2	5	2
13	5	2
12.8	5	2

12	5	2
10.6	5	2
12	5	2
13.95	5	2
13.8	5	2
13.4	5	2
11.5	5	2
12.2	5	2
10.65	5	2
9.7	5	2
12.6	5	2
10.7	5	2
13.6	6	2
9.1	5	2
13.55	6	2
11	5	2
10.55	5	2
11.8	5	2
13.05	5	2
10.75	5	2
12	5	2
10.4	5	2
11.25	5	2
9.95	5	2
11.35	5	2
9.8	5	2
11.1	5	2
12.3	5	2
10.75	5	2
11.85	5	2
10.4	5	2
9.45	5	2
9.1	5	2
10.4	5	2
11.05	5	2
11.1	5	2
9.3	5	2
14.55	5	2
14.5	5	2
13.45	5	2
12.2	5	2
13	5	2
14.5	5	2
13.95	5	2
12.5	5	2
11.55	5	2
13.35	5	2

12.85	5	2
10.7	4	2
13.4	5	2
13.7	5	2
11.65	5	2
11.8	5	2
12.4	5	2
14	5	2
11	5	2
12.5	5	2
14.8	5	2
13.7	5	2
13.05	5	2
13.75	5	2
13.85	5	2
14.15	5	2
13.05	5	2
15.3	5	2
11.65	5	2
12	5	2
13.65	5	2
12.25	5	2
13.4	5	2
12.55	5	2
13.85	5	2
12.65	5	2
9.65	5	2
13.65	5	2
12.9	5	2
11.35	5	2
11.1	5	2
14.05	5	2
13	5	2
13.3	5	2
11.05	5	2
12.8	5	2
13.05	6	2
10.9	4	2
11.9	5	2
12.9	5	2
11.8	5	2
11.3	4	2
9.45	4	2
11.45	5	2
11.75	5	2
12	5	2
14.3	5	2

12.3	5	2
14.8	5	2
14.85	5	2
10.95	5	2
10.1	5	2
9.75	4	2
8.6	4	2
11	5	2
12.5	5	2
9.4	5	2
9.8	5	2
12.05	5	2
10.3	5	2
12.01	5	2
11.75	5	2
11.01	5	2
9.15	5	2
7.45	5	2
11	5	2
11.35	5	2
9.65	5	2
11.25	5	2
10.65	5	2
15.25	5	2
13.75	5	2
16.5	5	2
16.45	5	2
17.05	5	2
16.8	5	2
17	5	2
19.5	5	2
15.6	5	2
15.6	5	2
15	5	2
17.2	5	2
16.4	5	2
17.65	5	2
17.2	5	2
17.55	5	2
17.95	5	2
17.1	5	2
15.95	5	2
16.5	5	2
12	5	2
11.75	6	2
11	5	2
9.15	5	2

7.45	5	2
11	4	2
11.35	5	2
9.65	5	2
11.25	5	2
10.65	5	2
15.25	5	2
13.75	5	2
16.55	5	2
16.45	5	2
17.05	5	2
16.8	5	2
17	5	2
19.5	5	2
15.6	5	2
15.6	5	2
9	6	2
10.2	5	2
10.4	5	2
10.8	5	2
10.7	6	2
9.8	5	2
9.45	5	2
10	6	2
9.5	5	2
10	5	2
10.05	5	2
9.35	4	2
10	5	2
11.55	5	2
10.7	6	2
9.65	4	2
10.85	5	2
12.25	5	2
11.45	6	2
10.7	5	2
15.8	5	2
14.15	5	2
13.9	5	2
14.3	5	2
11.65	5	2
10.75	5	2
10.75	5	2
12.5	5	2
11.7	5	2
11.4	5	2
11.85	5	2

12.3	5	2
11.6	5	2
11.25	5	2
13.2	5	2
12.45	5	2
12.95	5	2
14.45	5	2
12.1	5	2
11.05	5	2
14.35	5	2
14.9	5	2
15.85	6	2
15.1	5	2
16.05	5	2
15.15	5	2
17.15	5	2
15.85	6	2
15.35	6	2
15.85	5	2
15.45	5	2
13.15	5	2
14.9	6	2
14.05	5	2
13	5	2
14.3	5	2
18.4	6	2
13.5	5	2
14.1	5	2
17.85	6	2
14.5	5	1
13.9	5	1
16.3	5	1
13.4	5	1
16.4	5	1
15.3	5	1
15.4	5	1
15.5	5	1
13.4	5	1
13.8	5	1
15.7	5	1
12.4	5	1
14.95	5	1
13.2	5	1
14.8	4	1
11.95	5	1
14.7	5	1
15.5	5	1

13.45	5	1
14.7	5	1
10.5	5	1
9.8	4	1
12.85	5	1
14.6	5	1
14.2	5	1
13.3	5	1
13	5	1
14.25	5	1
14.15	5	1
12.7	5	1
12.55	5	1
12.75	5	1
16.05	5	1
10.65	5	1
12	5	1
13.25	5	1
12.55	5	1
15.8	5	1
11.25	5	1
13.8	5	1
10.1	5	2
9.55	5	2
11.35	5	2
10	5	2
10.95	6	2
10.4	5	2
9.5	5	2
10.85	5	2
10	5	2
6.75	4	2
10	5	2
10.85	5	2
9.05	4	2
10.7	5	2
11.2	5	2
10	4	2
11.6	5	2
10	5	2
8.7	5	2
8.4	5	2
8.05	5	2
9	5	2
9.4	5	2
10.1	5	2
9.4	5	2

9.85	5	2
9.05	5	2
8.85	5	2
9.7	4	2
10.25	5	2
7.65	5	2
9.15	5	2
9.2	5	2
9.1	5	2
8.65	6	2
9.1	5	2
8.55	4	2
8.05	5	2
9.25	5	2
7.55	5	2
10	5	2
10.85	5	2
9.05	4	2
10.7	5	2
11.2	5	2
10	4	2
11.6	5	2
10	5	2
8.7	5	2
8.4	5	2
8.05	5	2
9	5	2
9.4	5	2
10.1	5	2
9.4	5	2
9.85	5	2
9.05	5	2
8.85	5	2
9.7	4	2
10.25	5	2
14.4	5	3
15.1	5	3
13.9	5	3
13.1	5	3
13.55	5	3
14	5	3
13.8	5	3
12.7	5	3
12.35	5	3
10.9	5	3
13.1	5	3
13.7	5	3

16.55	5	3
13.1	5	3
11.9	5	3
13.85	5	3
12.45	5	3
13	5	3
14.1	5	3
14.95	5	3
12.35	5	3
12.9	5	3
14.8	5	3
14.55	5	3
12.6	5	3
13.6	5	3
13.6	5	3
13.6	6	3
12.95	6	3
12.75	5	3
14.85	5	3
14.45	5	3
15.5	5	3
16	5	3
15.9	5	3
14	5	3
15.4	5	3
12.05	5	3
11.75	5	3
16	5	3
13.7	5	3
14.2	5	3
11.95	5	3
11.4	5	3
12.65	5	3
13.3	5	3
11.6	5	3
11.45	5	3
10.7	5	3
11.05	5	3
12.8	6	3
13.35	5	3
11.5	5	3
10.85	5	3
12.5	5	3
11.65	5	3
10.85	5	3
13.3	5	3
11.3	5	3

12.8	5	3
10.45	4	2
13	5	2
10.55	4	2
11.85	4	2
10.4	5	2
13	5	2
12	5	2
9.55	4	2
9.9	4	2
13.8	5	2
12.55	5	2
13.15	5	2
15.35	5	2
14.2	5	2
15.5	5	2
11.5	5	2
12.8	5	2
11	5	2
11.7	5	2
13.65	5	2
10.9	5	2
10.8	5	2
11.15	5	2
12.2	5	2
10.9	5	2
10.55	5	2
10.5	4	2
12	5	2
10.65	5	2
10.6	5	2
10.75	5	2
10.45	5	2
10.9	5	2
9.85	5	2
10.05	5	2
8.9	5	2
11.65	5	2
11.4	5	2
10	5	2
11.25	5	2
13.55	5	2
13.9	5	2
16.5	5	2
14.45	5	2
14	5	2
14.75	5	2

14.65	5	2
14.6	5	2
12	5	2
12.25	5	2
13.4	5	2
11.8	5	2
12.3	5	2
14.5	5	2
13.3	5	2
12.7	5	2
13.3	5	2
12.8	5	2
13.75	5	2
8.75	5	2
11.75	5	2
10.9	5	2
10.75	5	2
11.5	5	2
11.2	5	2
12.3	6	2
13.4	5	2
11.75	5	2
10.2	5	2
10.65	5	2
13.55	6	2
11.2	6	2
11.45	4	2
12.8	5	2
12.1	5	2
11.25	6	2
12.05	5	2
11.3	5	2
11.6	5	2
12.55	5	2
12.15	5	2
11	5	2
12	5	2
11.05	5	2
11.6	5	2
11.3	5	2
11.3	5	2
14.55	6	2
11.55	5	2
13.85	5	2
10.9	5	2
12.05	5	2
11.7	6	2

12.65	5	2
11.8	6	2
12.8	5	2
11.6	5	2
12.3	5	2
14.3	7	2
14.2	6	2
11	6	2
12.15	5	2
11.3	5	2
13.5	5	2
11.4	5	2
10.95	5	2
7.5	5	2
12.6	6	2
13	6	2
11.35	5	2
11.6	5	2
9.9	5	2
10.25	5	2
11.1	5	2
11.85	5	2
13.75	6	2
11.05	6	2
11.45	6	2
12.5	6	2
16.05	6	2
15.9	5	3
14.7	5	3
12.35	5	3
14.15	5	3
13.3	5	3
15.3	5	3
15.25	5	3
17.15	5	3
19.25	5	3
16.3	6	3
14.7	5	3
19.3	5	3
15.7	5	3
14.05	5	3
15.85	5	3
12.9	4	3
15.15	4	3
15.6	5	3
17.8	5	3
17.7	5	3

15.6	5	3
13.05	5	3
15.15	5	3
13.25	5	3
14.55	5	3
15.35	5	3
12.7	5	3
13	5	3
13.1	5	3
14.9	5	3
15.1	5	3
14.45	5	3
12	5	3
12.45	5	3
12.5	5	3
11.65	5	3
15.8	5	3
12.05	5	3
13.05	5	3
14.7	5	3
15.55	5	3
13.7	5	3
14.95	5	3
17.1	6	3
14.3	5	3
13	5	3
13	5	3
16.3	5	3
15.9	5	3
14.15	5	3
12.45	5	3
13.65	5	3
12.45	5	3
15.5	6	3
16.4	6	3
13.7	5	3
13.55	5	3
13.8	5	3
14	5	3
18.05	5	3
6.1	5	2
7.7	5	2
8.6	5	2
6.1	6	2
6.4	5	2
7.25	5	2
8	5	2

9.05	5	2
6.95	5	2
8.3	5	2
6.65	5	2
8.55	5	2
7.45	5	2
7.4	5	2
7.2	5	2
7.7	5	2
7.6	5	2
8.3	5	2
8.3	5	2
6.9	5	2
8.25	5	2
7.65	5	2
7.95	5	2
8.65	5	2
9.4	5	2
9.15	5	2
7.8	5	2
9.35	5	2
8.75	5	2
7.3	5	2
9.5	5	2
9.8	5	2
8.3	5	2
9.8	5	2
9.45	5	2
8.05	5	2
8.05	5	2
9.4	5	2
9.4	5	2
9.15	5	2
11	5	2
8.35	5	2
9.05	5	2
8.95	5	2
8.15	5	2
8	5	2
8.4	5	2
9.8	5	2
9.6	5	2
9.85	5	2
9.2	5	2
9.9	5	2
8.1	5	2
9.75	5	2

9.85	5	2
10	5	2
9.85	5	2
8.85	5	2
9.45	5	2
8.75	5	2
12.15	5	2
10.45	5	2
12.3	5	2
10.75	5	2
11.95	5	2
10.35	5	2
12.1	5	2
12.05	5	2
11.2	5	2
10.3	5	2
12.7	5	2
12.1	5	2
11.05	5	2
12.55	5	2
12.15	5	2
10.4	5	2
12.6	5	2
11.3	5	2
10.45	5	2
11.9	5	2
11.25	5	2
11.2	5	2
10.95	5	2
9.95	5	2
11.05	5	2
11.3	5	2
12.25	5	2
11.05	5	2
10.35	5	2
10.6	5	2
10.25	5	2
10.75	5	2
10.4	5	2
11.3	5	2
10.4	5	2
11.1	5	2
11.5	5	2
10.9	5	2
11.2	5	2
10.35	5	2
10.7	5	2

10.45	5	2
10.7	5	2
10.2	5	2
9.7	5	2
12.55	5	2
11.2	5	2
11.4	5	2
9.95	5	2
11.95	5	2
9.15	5	2
11.8	5	2
9.1	5	2
11	5	2
10.3	5	2
11	5	2
10.9	5	2
10.95	5	2
10.45	5	2
11.1	5	2
7.55	5	1
10.25	5	1
9.8	5	1
11.95	5	1
10.6	5	1
9.8	5	1
11	5	1
11.2	5	1
13.25	7	1
8.6	5	1
8.8	5	1
9.7	5	1
9.9	5	1
9.3	5	1
9.9	5	1
9.8	5	1
10	5	1
9.6	5	1
8.8	5	1
9.05	5	1
12.05	5	3
12	5	3
10.9	5	3
11.35	5	3
13.5	4	3
12.9	5	3
13.95	5	3
10.35	5	3

10.8	5	3
12.45	5	3
12	5	3
11.7	5	3
11.1	5	3
12.1	5	3
11.55	5	3
9.85	5	3
11.2	5	3
11	5	3
12.85	5	3
11.7	5	3
13.6	5	3
12.55	5	3
13.25	5	3
10.45	5	3
12.3	5	3
14.5	5	3
13.95	5	3
11.9	5	3
13.45	5	3
13.2	5	3
14.85	5	3
14.8	5	3
11.95	5	3
15.05	5	3
12.85	5	3
14.25	5	3
15.4	5	3
14.3	5	3
13.9	5	3
15.7	5	3
13.15	5	3
12	5	3
14.1	5	3
14.65	5	3
10.25	5	3
14.7	5	3
13.05	5	3
10.7	5	3
14.2	5	3
12.45	5	3
14.95	5	3
14.55	5	3
12.8	5	3
13.9	5	3
14.5	5	3

14.95	5	3
12.3	5	3
12.9	5	3
12	5	3
12.05	5	3
8.7	5	3
9.3	5	3
8.7	5	3
8.95	5	3
7.8	5	3
10	5	3
8.75	5	3
8.6	5	3
8.25	5	3
9.1	5	3
7.75	5	3
7.75	5	3
9.25	5	3
9.75	5	3
8.7	5	3
8	5	3
9.4	5	3
8.85	5	3
8.85	5	3
8.45	5	3
10.5	5	3
10.75	5	3
10.8	5	3
9.8	5	3
9	5	3
9	5	3
10.05	5	3
9.65	5	3
9.55	5	3
11.1	5	3
11	5	3
10.1	5	3
10	5	3
10.45	5	3
10.55	5	3
11.9	5	3
10.1	5	3
11.35	5	3
11.35	5	3
10.05	5	3
9.8	5	3
11.3	5	3

12.8	5	3
11.8	5	3
11.55	5	3
10.2	5	3
10	5	3
9.9	5	3
10.05	5	3
10	5	3
9.5	5	3
9.6	5	3
10.4	5	3
10.5	5	3
8.75	5	3
10.55	5	3
8.8	5	3
8.7	5	3
9.05	5	3
11.35	5	3
12.1	5	3
11.65	5	3
13.2	5	3
12.85	5	3
12.85	5	3
13.4	5	3
10.8	5	3
13.4	5	3
12.7	5	3
13	5	3
12.55	5	3
12.9	5	3
13.95	5	3
16.9	6	3
13.4	5	3
12.8	5	3
12.2	5	3
12.45	5	3
13.25	5	3
13.25	5	3
11.35	5	3
11.05	5	3
11.1	5	3
10.35	5	3
11.3	5	3
12.45	5	3
10.9	5	3
11.25	5	3
12.1	5	3

11.75	5	3
10.45	5	3
11.35	5	3
10.4	5	3
10.4	5	3
10.9	5	3
12.15	5	3
10.95	5	3
10.85	5	3
9.5	5	3
12	5	3
10.5	5	3
13.45	5	3
10.1	5	3
11.5	5	3
12.65	5	3
12.8	5	3
11.45	5	3
13.4	5	3
10.1	5	3
12.2	5	3
11.95	5	3
11.2	5	3
10.4	5	3
13.7	5	3
10.15	6	3
12.4	5	3
12.35	5	3
10.5	5	3
9.95	5	3
9.5	5	3
16.7	5	3
15.7	5	3
16.9	5	3
15	5	3
15.8	5	3
17.05	5	3
13.7	5	3
16.05	5	3
14.2	5	3
17.7	5	3
17.45	5	3
14.7	5	3
15.7	5	3
16.35	5	3
18.45	5	3
19.2	6	3

16.55	5	3
15.1	5	3
17.9	5	3
14.4	5	3
13.3	6	3
15.9	6	3
14.5	5	3
13.1	5	3
13.45	6	3
14.4	5	3
15.9	6	3
13.35	5	3
15.7	5	3
14.4	5	3
12.7	6	3
13.5	5	3
14.25	6	3
15	6	3
14.55	5	3
14.5	5	3
11.95	5	3
15.35	6	3
12.6	5	3
12.9	5	3
9.3	5	2
8.55	5	2
9.2	5	2
7.95	5	2
9.6	5	2
8.7	5	2
8.25	5	2
7.95	5	2
8.95	5	2
9.15	5	2
8	5	2
7.95	5	2
8.1	5	2
10.2	5	2
9.7	5	2
8.5	5	2
8.55	5	2
9.4	5	2
8.65	5	2
8.85	5	2
7.9	5	2
10.4	5	2
10.3	5	2

11.35	5	2
10.7	5	2
8.95	5	2
9.05	5	2
9.65	5	2
11.05	5	2
8.95	5	2
8.9	5	2
10.8	5	2
9.55	5	2
10.2	5	2
10.45	5	2
9.7	5	2
9.05	5	2
10.8	5	2
10	5	2
8.3	4	2
7.5	5	2
9.2	4	2
9.9	5	2
7.5	5	2
10.7	5	2
10.25	5	2
8.95	5	2
10	5	2
8.15	5	2
9.1	5	2
9.75	5	2
8.8	5	2
9.8	5	2
11	5	2
8.8	5	2
9	5	2
8.95	5	2
9.3	5	2
10.1	5	2
10.55	5	2
13.05	5	2
12.55	5	2
12	5	2
12.95	5	2
10.7	5	2
11.6	5	2
11.7	5	2
13.15	5	2
12.05	5	2
12.6	5	2

13.2	5	2
12.75	5	2
12.45	5	2
12.75	5	2
12.85	5	2
10.65	5	2
10.75	5	2
12.4	5	2
12	5	2
13.15	5	2
11.55	5	2
12.05	5	2
12	5	2
13	5	2
11.55	5	2
12.3	5	2
13.45	5	2
13.75	5	2
10.75	5	2
12.9	5	2
13.85	5	2
13.2	5	2
16.05	5	2
13.9	6	2
12.3	5	2
12	5	2
13.25	5	2
13.7	5	2
12.9	5	2
13.65	5	2
12.4	5	2
11.95	5	2
13.1	5	2
12.5	5	2
13.05	5	2
11.3	5	2
11.7	5	2
13.4	5	2
14.2	5	2
14.55	5	2
13.8	5	2
14.5	5	2
14.6	5	2
13.15	5	2
10.8	5	2
9.95	5	2
12.7	5	2

12.3	5	2
12.15	5	2
14.55	5	2
19.05	6	5
13.5	5	5
16.9	5	5
14.7	5	5
18.95	6	5
14.4	5	5
14.35	5	5
17.8	6	5
14.6	5	5
18.1	5	5
16.7	5	5
16.15	5	5
15.55	5	5
13.75	5	5
16.1	6	5
14.2	5	5
14.15	5	5
15.4	5	5
14.6	5	5
16.5	5	5
13.4	5	5
14.15	5	5
14.75	5	5
15.45	5	5
10.25	5	5
11.65	4	5
12.95	5	5
15.6	5	5
14.95	5	5
14.5	5	5
15.05	5	5
13.6	5	5
13.55	5	5
13	5	5
13.85	5	5
12	5	5
13.65	5	5
12.6	5	5
13.45	5	5
15.85	6	5
12.3	5	5
10.9	5	5
10.9	4	5
12.4	5	5

12.1	5	5
13.85	5	5
12.7	5	5
13.4	5	5
13.4	5	5
11.95	5	5
13.45	5	5
12.05	5	5
11.4	5	5
13.25	5	5
11.65	5	5
14	5	5
13.75	5	5
12.65	5	5
12.5	5	5
12.5	5	5
12.55	6	2
10.95	5	2
10.05	5	2
11.5	5	2
10.8	6	2
10.75	5	2
12.95	5	2
11.3	5	2
11.8	5	2
10.85	5	2
11.1	5	2
10.15	5	2
9.95	5	2
11.05	5	2
11.5	5	2
11.3	5	2
10.85	5	2
12	5	2
10.8	5	2
12.5	5	2
11.15	5	2
10.1	5	2
10.35	5	2
10.25	5	2
10.65	5	2
9.9	5	2
10.35	5	2
11.1	5	2
9	4	2
10.85	5	2
10.7	5	2

10	5	2
10.25	5	2
10.75	5	2
8.45	5	2
10.5	5	2
9.6	5	2
11.3	5	2
9.6	5	2
10.95	5	2
11.05	6	2
11.2	5	2
11.5	5	2
10.05	5	2
10.5	5	2
10.75	5	2
10.7	5	2
10.15	5	2
11.4	6	2
12.9	6	2
11	5	2
10.1	5	2
10.95	5	2
9.85	5	2
12.05	6	2
10.6	5	2
9.65	5	2
11.45	6	2
11	5	2
10.9	6	2
11.25	5	1
12.45	5	1
11.7	5	1
10.55	5	1
9.7	5	1
11	5	1
10.55	5	1
10.75	5	1
9.7	5	1
11.1	5	1
10.95	5	1
12.3	5	1
10	5	1
10.95	5	1
11	5	1
13.45	5	1
10.1	5	1
12.3	5	1

14.7	5	1
10.85	5	1
10.85	5	1
12.2	5	1
11.9	5	1
11.1	5	1
12.15	5	1
13.9	6	1
12.05	5	1
10.65	5	1
13.15	5	1
10.95	5	1
11.3	5	1
11.05	5	1
10.95	5	1
10.5	5	1
11.5	5	1
13.55	5	1
11.4	5	1
12.7	5	1
11.1	5	1
13.4	5	1
10.5	5	1
9.95	5	1
9.7	5	1
10.2	5	1
13.8	7	1
14.1	7	1
10.95	6	1
9.8	5	1
10.65	5	1
10.35	6	1
9.65	5	1
10.5	5	1
9.55	6	1
9.75	6	1
9.45	5	1
9.7	5	1
10	6	1
7.9	5	1
8.7	6	1
8.3	5	1
9.7	5	1
9.7	4	1
9	5	1
9.95	5	1
10.35	5	1

10.5	5	1
9.75	4	1
10	5	1
10	5	1
8.55	5	1
4.9	5	1
7.05	4	1
9.05	5	1
6.7	5	1
9.4	5	1
10.6	5	1
10.1	5	1
9.3	6	1
10.5	5	1
7.9	5	1
10.9	6	1
11.8	5	1
12.2	6	1
12.45	6	1
13.8	5	1
11.2	5	1
11.85	5	1
10.45	5	1
12.05	5	1
10.95	5	1
12.1	5	1
10.25	4	1
10.75	5	1
10.15	6	1
10.45	5	1
11.35	5	1
10.5	6	1
11.7	5	1
10.85	5	1
12.65	6	1
11.55	5	2
12.25	5	2
13.9	5	2
14.25	5	2
13.8	4	2
9.5	5	2
15.2	5	2
12.55	5	2
14.8	5	2
14.05	5	2
12.6	5	2
14.05	5	2

13.65	5	2
11.85	5	2
10.95	5	2
14.15	5	2
11	5	2
13.5	5	2
14.75	5	2
9.3	5	2
13.05	5	2
13.1	5	2
11.4	5	2
12.8	5	2
12.6	5	2
15.6	5	2
15.75	5	2
13.15	5	2
14.1	5	2
14.35	5	2
13.65	5	2
13.5	5	2
14.6	5	2
15.7	5	2
13.95	5	2
13.85	5	2
13.55	5	2
13.6	5	2
14.8	5	2
14.25	5	2
14.1	5	2
13.85	5	2
15.6	5	2
12.45	5	2
13.35	5	2
15.55	5	2
16.4	5	2
14.75	5	2
16.8	6	2
16.9	6	2
11.5	5	2
14.4	5	2
14.5	5	2
13.05	5	2
13.75	5	2
11.1	5	2
11.8	5	2
14.15	5	2
12.6	5	2

17.1	6	2
12.35	5	2
12.65	5	2
12.9	5	2
13.4	5	2
13.95	5	2
13.95	5	2
11.3	5	2
11.75	6	2
10.85	5	2
12.5	6	2
9.6	5	2
10.3	5	2
11.95	5	2
9.4	5	2
10.05	5	2
30.6	5	2
10.15	5	2
12.4	5	2
8.9	5	2
8.25	5	2
8.95	5	2
12.05	5	2
10.85	5	2
8.25	5	2
9.9	5	2
8.8	5	2
9	5	2
10.4	5	2
8.75	5	2
10	5	2
10.1	5	2
9.4	5	2
8.95	5	2
9.6	5	2
11.9	5	1
10.05	5	1
10.4	5	1
11.45	5	1
11.8	5	1
10.55	5	1
9.35	5	1
12.2	5	1
11.35	5	1
11.5	5	1
10.7	5	1
13.2	5	1

12.5	5	1
11.65	5	1
10.45	5	1
11.65	5	1
12.7	5	1
12.5	6	1
11.35	5	1
10.8	5	1
11.9	5	1
10.9	5	1
10.55	5	1
9.25	5	1
10.9	5	1
11.5	5	1
11	5	1
11.05	5	1
11.2	5	1
9.55	5	1
11.25	5	1
11	5	1
11.1	5	1
10.5	4	1
11.3	5	1
9.35	5	1
11.9	5	1
11.65	5	1
10.9	5	1
10.8	5	2
11.05	5	2
8.3	6	2
8.3	6	2
11.2	5	2
8.8	5	2
9.5	5	2
9.1	5	2
9.95	5	2
9.15	5	2
8.8	5	2
11.65	7	2
9.4	6	2
9.9	6	2
8.8	5	2
8.5	5	2
9.65	5	2
9.65	6	2
7.6	5	2
8.7	6	2

8.65	5	4
9.95	5	2
10.9	5	2
9.85	5	2
9.55	5	2
7.2	5	2
11.5	5	2
7.15	5	2
10.4	5	2
8	5	2
9.65	5	2
10.95	5	2
11.55	5	2
11.25	5	2
12.1	5	2
10.8	5	2
9.6	5	2
8.4	5	2
9.3	5	2
7	5	2
9.95	5	2
13.1	5	2
14.8	5	2
12.55	5	2
11.85	5	2
10.85	5	2
12.8	5	2
12.9	5	2
12.45	5	2
14.1	5	2
13.6	6	2
13.65	5	2
11.35	7	2
14.85	6	2
14.3	5	2
10.5	5	2
12.5	6	2
13.1	5	2
12.8	5	2
12.25	5	2
11.1	5	2
12	5	2
11.65	5	2
12.45	5	2
13.25	6	2
12.3	6	2
10.4	5	2

12.55	5	2
11.45	5	2
14.55	5	2
17.4	5	2
15.65	5	2
13.9	5	2
14.6	5	2
18.35	6	2
16	5	2
17.45	5	2
18.85	5	2
15.85	5	2
14.4	6	2
13.2	5	2
14.15	5	2
5.8	5	2
9.1	5	2
11.1	6	2
9.35	2	2
10.2	5	2
8	5	2
8.55	5	2
8.7	5	2
6.6	5	2
8.8	5	2
6.6	6	2
9.5	5	2
7.05	5	2
6.55	5	2
7.2	5	2
8.45	5	2
5.55	5	2
7.3	5	2
6.35	5	2
8.55	6	2
5.75	5	2
9.25	5	2
7.4	5	2
7.9	5	2
6.65	5	2
13	5	2
14.9	5	2
13.2	5	2
10.5	5	2
14.5	5	2
13.95	6	2
13.35	5	2

12.2	5	2
11.6	5	2
12.2	5	2
11.85	5	2
11.55	5	2
11.2	5	2
7.3	5	2
12.35	5	2
9.95	5	2
11.6	5	2
11.1	5	2
11.7	5	2
11.7	5	2
12.05	5	2
13	5	4
12.8	5	4
14.6	5	4
13.25	5	4
12.35	5	4
11.7	5	1
12.8	5	1
10.95	5	1
11.1	5	1
12.7	5	4
12.6	5	4
13.15	5	4
12.35	5	4
13.05	5	4
11.15	6	4
12.8	5	4
10.15	5	1
8.05	5	1
10.1	5	1
9.85	5	1
10.6	5	6
9.9	5	2
9.4	5	1
11.2	5	1
10.6	5	1
10.75	5	1
9.5	5	1
9.5	5	1
9.7	5	1
9.4	5	1
8.65	5	1
9.45	5	6
11	5	6

10.15	5	2
12.5	5	2
12.35	5	2
11	5	2
7.25	5	2
9.25	5	2
8.05	6	5
9.15	5	5
8.95	5	5
9.55	5	6
10.5	5	6
9.85	5	6
9.05	5	6
10.55	5	6
10.25	5	6
10.8	6	1
11.3	5	1
9.9	4	1
8.9	5	1
8.25	5	1
10.45	5	1
8.05	4	1
9.9	5	1
10.1	6	1
10.3	5	1
13.9	7	1
10	6	1
9.9	5	1
8.7	4	1
7.6	4	1
10.4	5	1
9.7	5	1
9.9	5	1
9.05	5	1
10.65	5	1
11.65	5	2
11.1	5	2
11.7	5	2
12.55	5	2
12.35	5	2
9.75	5	2
10.95	5	2
7.7	5	2
8.15	5	2
8.05	5	2
10.1	5	2
11.95	5	2

11.1	6	2
11.2	5	2
11.45	5	2
13.45	5	2
12.75	5	2
10.65	5	2
10.45	5	2
10.5	5	2
12.35	5	2
10.2	5	2
11.5	5	2
11.4	5	2
10.2	5	2
10.85	4	2
12.1	5	2
10.25	5	2
10.55	5	2
10.8	5	2
10	5	2
9.35	6	2
9.7	6	2
10.4	5	2
8.4	5	2
8.5	5	2
8.1	5	2
8.9	5	2
8.3	5	2
9.1	5	2
8.3	5	2
9.85	5	2
7.75	4	2
8.7	5	2
8.7	5	2
7.75	5	2
8.75	5	2
8.75	5	2
8.65	5	2
8.7	5	2
11.6	5	2
8.65	5	2
8.95	5	2
8.7	5	2
8.8	5	2
9.7	5	2
11.65	6	2
9.9	5	2
9.65	6	2

9.25	5	2
9	5	2
9.05	5	2
7.65	5	2
7.4	4	2
8.65	4	2
9.4	4	2
9.1	5	2
9	5	2
7.85	5	2
10.1	5	2
10.5	5	2
10.5	5	2
9.7	5	2
9.65	6	2
9.15	5	2
10.6	5	2
10.7	5	2
10.1	5	2
8.8	5	2
10.05	5	2
10.3	5	2
10	5	2
10.7	5	2
8	5	2
8.6	5	2
9.8	5	2
9.9	5	2
9.55	5	2
9.6	5	2
8.1	5	2
10.2	5	2
10.1	5	2
10.1	5	2
10.65	5	2
10.05	5	2
9.1	5	2
8.95	5	2
10	5	2
10.05	5	2
10.25	5	2
10.9	5	2
10.3	5	2
9.05	5	2
11	5	2
11.05	6	2
17.4	9	2

8.25	5	2
8.85	5	2
9.55	5	2
12	6	2
9.05	5	2
9.4	5	2
9.5	5	2
10.35	5	2
9	5	2
9.5	5	2
10.2	5	2
10.35	5	2
