# Supplementary figures and images for: Flower, fruit phenology and flower traits in Cordia boissieri (Boraginaceae) from northeastern Mexico
Source: PeerJ. 2016 May 17;4:e2033. doi: 10.7717/peerj.2033 (PMC4878375; doi:10.7717/peerj.2033)

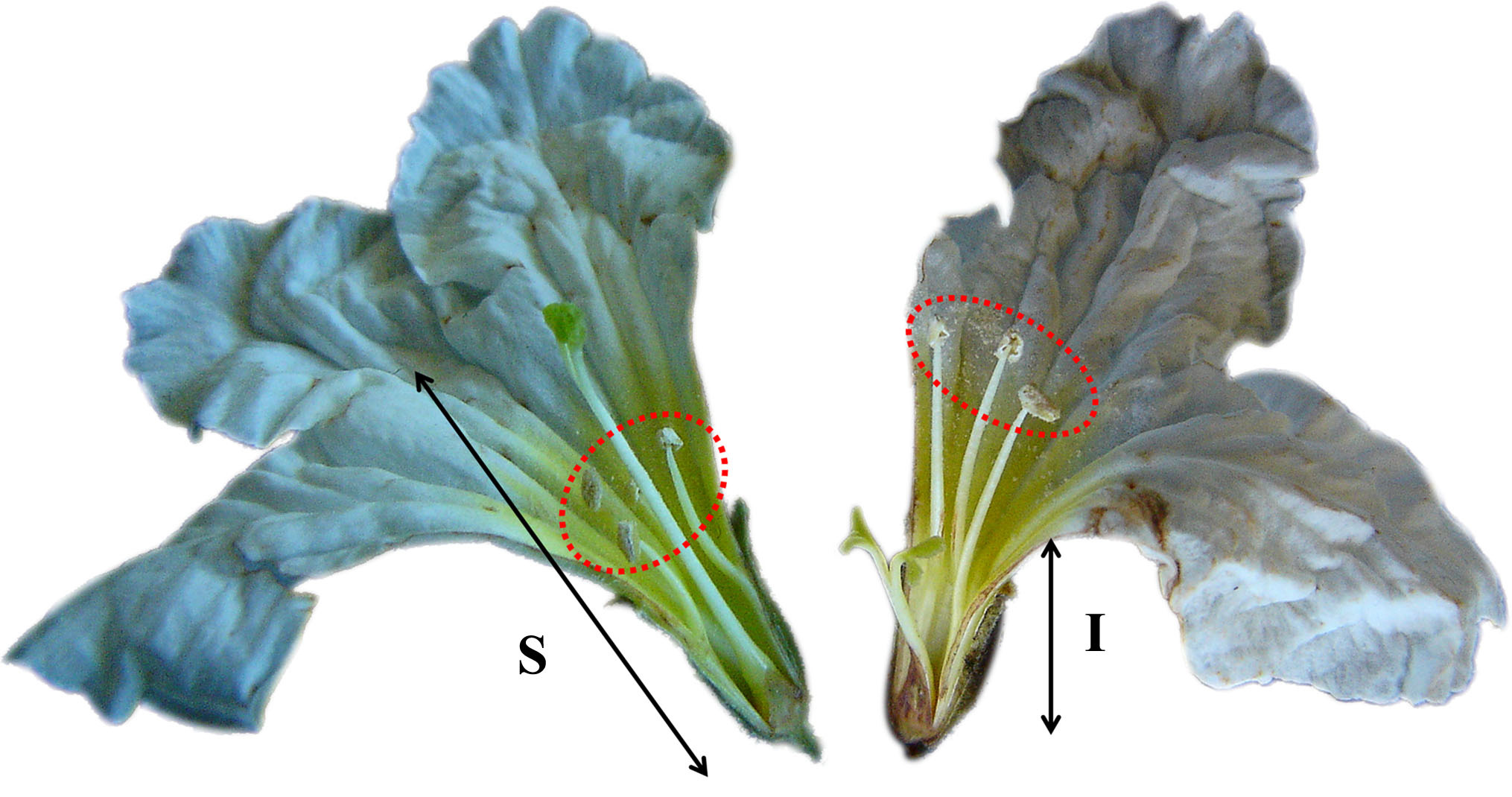

Supplement: Supplemental Information 2 — Arrows are parallel to the ovaries. S = superior style, I = inferior style. The stamens are highlighted inside dotted red lines. From 15,000 flowers (from 75 plants), we found 760 to have an inferior ovary (from 38 plants) and 740 to have a superior ovary (from 37 plants). [file peerj-04-2033-s002.jpg]

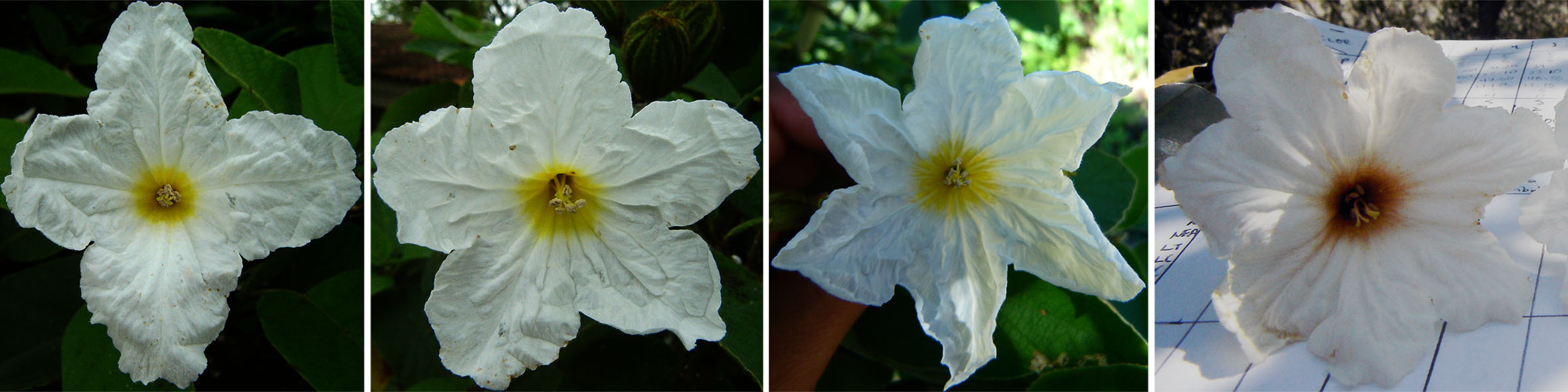

Supplement: Supplemental Information 3 [file peerj-04-2033-s003.jpg]

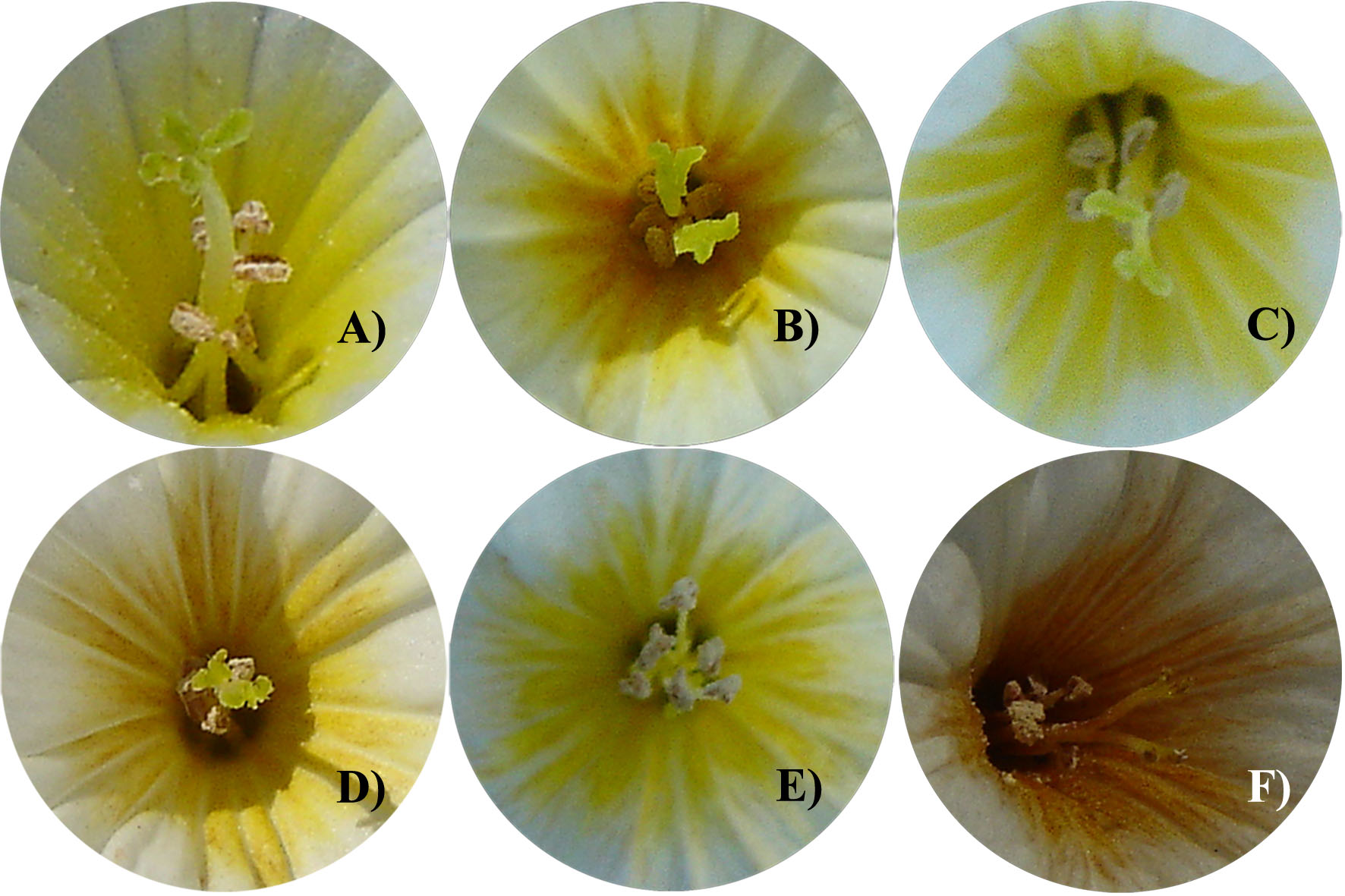

Supplement: Supplemental Information 4 [file peerj-04-2033-s004.jpg]
